# Smohaze‐Upregulated RFWD3 Competes with TRIM24 to Stabilize TREX1 and Reduce Cytosolic dsDNA in Non‐Small Cell Lung Cancer

**DOI:** 10.1002/advs.202508481

**Published:** 2025-10-21

**Authors:** Xue‐Yan Shi, Yu‐Ke Shen, Meng‐Yao Lv, Yu Sun, Yong‐Fang Lin, Zheng Wang, Xiao‐Liang Jie, Zheng Liu, Yang‐Tong Liu, Yang‐Xin Fu, Zhenhua Ren, Gui‐Zhen Wang, Guang‐Biao Zhou

**Affiliations:** ^1^ State Key Laboratory of Molecular Oncology National Cancer Center/National Clinical Research Center for Cancer/Cancer Hospital Chinese Academy of Medical Sciences and Peking Union Medical College Beijing 100021 China; ^2^ Institute of Cancer Research Henan Academy of Innovations in Medical Sciences Zhengzhou Henan 450000 China; ^3^ Tumor Immunology and Cytotherapy Medical Research Center The Affiliated Hospital of Qingdao University Qingdao Shandong 266000 China; ^4^ Changping Laboratory Beijing 102206 China; ^5^ State Key Laboratory of Molecular Oncology & Center for Cancer Biology School of Basic Medical Sciences Tsinghua University Beijing 100084 China; ^6^ Department of Lymphoma Peking University Cancer Hospital and Institute Beijing 100142 China

**Keywords:** cancer immunotherapy, cytosolic dsDNA, RFWD3, STING‐IFN, TREX1

## Abstract

The three‐prime repair exonuclease 1 (TREX1), an intracellular double‐stranded DNA (dsDNA) degrader that inhibits the stimulator of interferon (IFN) genes (STING) pathway, is turned over by E3 ligase TRIM24‐mediated proteasomal degradation. To uncover TREX1‐stabilizers in non‐small cell lung cancer (NSCLC), mass spectrometry is conducted, and 374 candidates are identified, with the RING Finger and WD Repeat Domain 3 (RFWD3) as a TREX1 protector that sequesters it from TRIM24. Overexpression of RFWD3 promotes tumor growth with increased myeloid‐derived suppressor cells (MDSCs), while inhibition of RFWD3 increases intracellular dsDNA levels, activates the STING‐IFN signaling, decreases MDSCs, and enhances the efficacy of PD‐L1 blockade in murine NSCLC models. Furthermore, smoker patients have higher RFWD3 levels than non‐smoker patients, and cigarette smoke extract, PM_2.5_, and benzo(a)pyrene upregulatesRFWD3 via transcription factor aryl hydrocarbon receptor. These results indicate a role of RFWD3 in tobacco smoke and haze (smohaze)‐promoted immune evasion, inhibition of which activates STING‐IFN signaling and synergizes with immune checkpoint inhibitors in NSCLC.

## Graphical abstract

The E3 ubiquitin ligase RFWD3 binds the exonuclease TREX1 and protects it from TRIM24‐mediated ubiquitination and degradation, leading to the eradication of intracellular dsDNA, inhibition of the cGAS‐STING pathway, and immunosuppressive microenvironment of lung cancer. Exposure to cigarette smoke and haze (PM_2.5_) upregulate RFWD3 via transcription factor AhR, while inhibition of RFWD3 enhances the therapeutic efficacy of PD‐L1 blockade, providing a potential approach to tackle smohaze‐induced lung cancer.

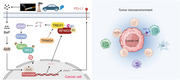

## 1 Introduction

Cancer cells are often replete with cytosol double‐stranded DNA (dsDNA),^[^ ^1^, ^2^ ^]^ which is able to activate the cyclic GMP‐AMP synthase (cGAS)‐stimulator of interferon (IFN) genes (STING) pathway to induce the production of type I interferon (IFN‐I).^[^ ^3^ ^]^ To inhibit this anti‐tumor immune reaction, cancer cells use exonucleases, mainly the 3′ – 5′ restriction exonuclease Three‐prime Repair Exonuclease 1 (TREX1), to degrade the abnormally accumulated cytosolic dsDNA and restrain the activation of cGAS‐STING cascade.^[^ ^4^, ^5^, ^6^ ^]^ Studies have shown that TREX1 is degraded by the E3 ubiquitin ligase Tripartite Motif Containing 24 (TRIM24), which is upregulated by wild‐type p53^[^ ^7^ ^]^ and is able to trigger ubiquitination and proteasomal degradation of p53^[^ ^8^ ^]^ and itself.^[^ ^9^ ^]^ TREX1 is inhibited by increased methylation and epigenetic silencing^[^ ^10^ ^]^ and DNA‐damaging agent‐induced miR‐103.^[^ ^11^ ^]^ However, the mechanisms of TREX1 stabilization and subsequent cGAS‐STING repression in cancers remain unclear.

RING Finger and WD Repeat Domain 3 (RFWD3, or RNF201, FANCW) is an E3 ubiquitin ligase that plays a crucial role in the DNA damage repair response.^[^ ^12^, ^13^, ^14^, ^15^ ^]^ Under replication stress and fork stalling, RFWD3 facilitates the restart of the stalled replication forks and homologous recombination at stalled forks.^[^ ^14^ ^]^ In contrast, RFWD3 dysfunction leads to unrepaired DNA damage and the initiation of diseases such as Fanconi anemia and multiple myeloma.^[^ ^12^, ^16^ ^]^ RFWD3 is overexpressed in bladder cancer,^[^ ^17^ ^]^ liver cancer,^[^ ^18^ ^]^ and colorectal cancer.^[^ ^19^ ^]^ We previously found that RFWD3 was overexpressed in patients with non‐small cell lung cancer (NSCLC), in particular those patients who smoked, and was associated with poor prognosis.^[^ ^20^ ^]^ However, the effects of RFWD3 on tumor microenvironment (TME) and signal pathways, including cGAS‐STING axis, still need to be determined.

In this study, we analyzed the interactome of TREX1 to identify proteins that can stabilize this exonuclease, and found that RFWD3 interacts with TREX1 and inhibits its degradation by competing with TRIM24 to repress TREX1 ubiquitination. Upregulation of RFWD3 promoted tumor growth and induced an immunosuppressive tumor microenvironment, while RFWD3 knockdown caused the accumulation of cytoplasmic dsDNA, which triggered the activation of STING‐IFN signaling to exert anti‐tumor immunity. Furthermore, air pollutants upregulated RFWD3 via activation of the transcription factor Aryl hydrocarbon receptor (AhR), suggesting the role of this E3 ligase in tobacco smoke and air pollution/haze (smohaze)‐induced immune escape and lung carcinogenesis.

## 2 Results

### 2.1 RFWD3 is Required for TREX1 Protein Stability and Exonuclease Activity

**Figure 1:**
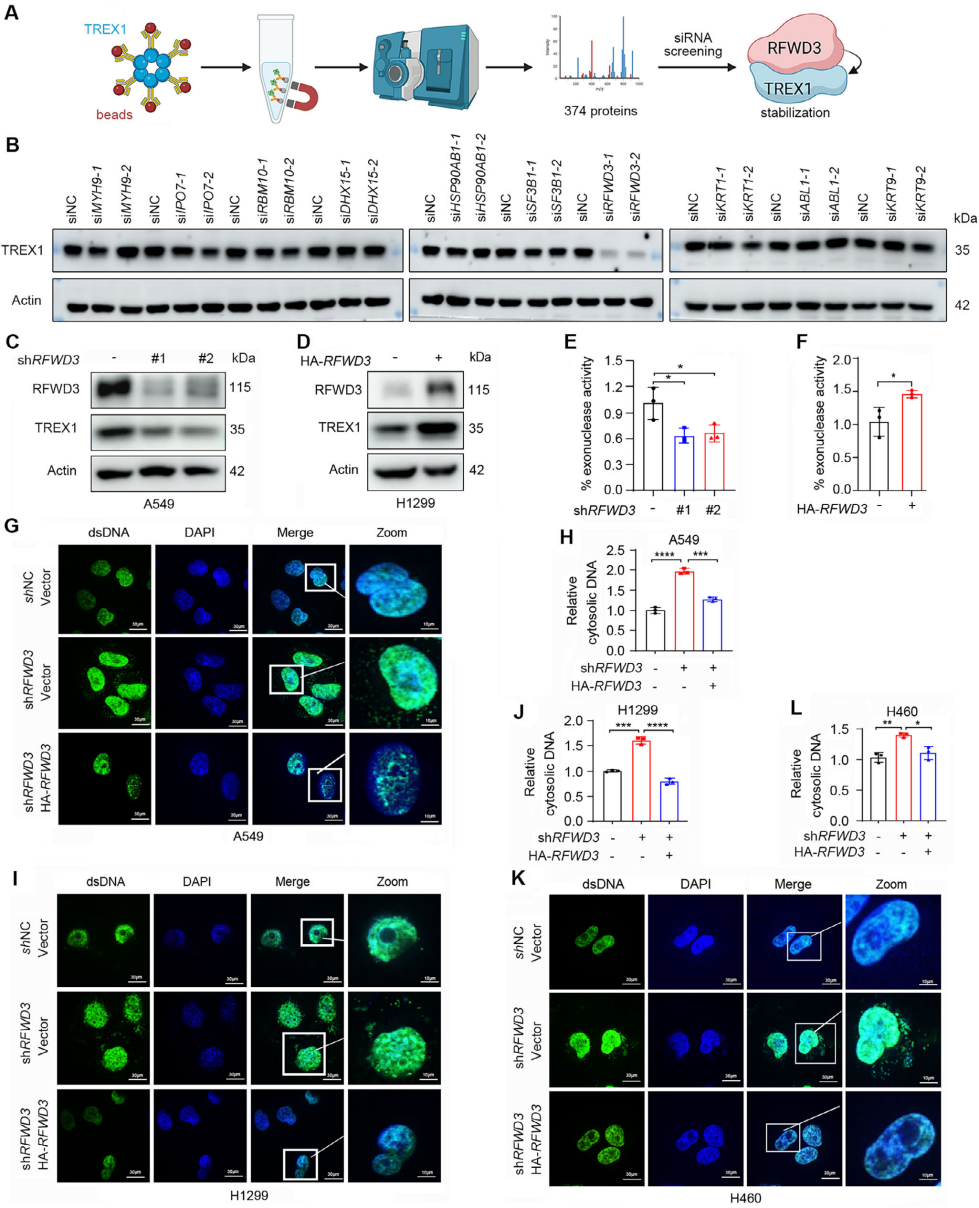
RFWD3 is required for TREX1 protein stability and exonuclease activity. A) Workflow for identification of TREX1‐protecting proteins. Flag‐*TREX1* or control plasmids were transfected into A549 cells, which were lysed 48 h later and subjected to immunoprecipitation using anti‐Flag magnetic beads to isolate TREX1 and its interacting proteins. Co‐precipitated proteins were analyzed by mass spectrometry (MS), identifying 374 potential TREX1‐binding candidates. Subsequent siRNA screening revealed RFWD3 as a key stabilizer of TREX1 protein. B) A549 cells were transfected with different siRNAs and subjected to western blotting. C) A549 cells were transfected with two different shRNAs for RFWD3 and subjected to western blotting. D) H1299 cells were transfected with HA‐*RFWD3* and subjected to western blotting. E) Representative graphs indicate % exonuclease activity in A549 cells transfected with two different shRNAs for RFWD3. F) Representative graphs indicate % exonuclease activity in H1299 cells transfected with HA‐*RFWD3*. G) Representative confocal microscopy images of cytosolic dsDNA in A549 cells detected using a dsDNA‐specific antibody. H) Cytosolic DNA was isolated and quantified from A549 cells stably expressing *RFWD3* shRNA and transfected with HA‐*RFWD3*. I) Representative confocal microscopy images of cytosolic dsDNA in H1299cells detected using a dsDNA‐specific antibody. J) Cytosolic DNA was isolated and quantified from H1299 cells stably expressing *RFWD3* shRNA and transfected with HA‐*RFWD3*. K) Representative confocal microscopy images of cytosolic dsDNA in H460 cells detected using a dsDNA‐specific antibody. L) Cytosolic DNA was isolated and quantified from H460 cells stably expressing *RFWD3* shRNA and transfected with HA‐*RFWD3*. Error bars, SD. *p*‐values were calculated using a two‐tailed Student's *t*‐test. ^*^, *p*< 0.05; ^**^, *p*< 0.01; ^***^, *p*< 0.001; ^****^, *p*< 0.0001.

To identify proteins that can stabilize TREX1, we first employed immunoprecipitation (IP) and mass spectrometry (MS) assays to uncover TREX1‐interacting proteins. A549 lung adenocarcinoma (LUAD) cells were transfected with either Flag‐TREX1 or an empty control vector. TREX1 protein was isolated using magnetic beads, and proteins from empty vector‐transfected cells served as controls to remove non‐specific interactions. The precipitated proteins were subjected to MS analysis, which identified 374 potential TREX1‐binding proteins (**Figure** **1**A). The top 10 candidates were Myosin Heavy Chain 9 (MYH9), Keratin 1 (KRT1), Importin 7 (IPO7), RNA Binding Motif Protein 10 (RBM10), DEAH‐Box Helicase 15 (DHX15), Heat Shock Protein 90 Alpha Family Class B Member 1 (HSP90AB1), Splicing Factor 3b Subunit 1 (SF3B1), RFWD3, KRT9, and Albumin (ALB) (Table S1, Supporting Information). We conducted small interfering RNA (siRNA; Table S2, Supporting Information)‐mediated gene silencing (Figure S1A–J, Supporting Information) to investigate the potential effects of these molecules on TREX1 protein stability. By western blot and densitometry analyses, we found that knockdown of *RFWD3*, *IPO7*, and *SF3B1* caused downregulation of TREX1 (Figure 1B; Figure S1K, Supporting Information). Since the effects of si*RFWD3* on TREX1 protein expression were much stronger than si*IPO7* and si*SF3B1* (Figure 1B; Figure S1K, Supporting Information), *RFWD3* was chosen for further investigation.

We further validated the effects of RFWD3 on TREX1, and found that silencing of RFWD3 by short hairpin RNA (shRNA) against RFWD3 (sh*RFWD3*; Table S2, Supporting Information) downregulated TREX1 (Figure 1C), while overexpression of RFWD3 upregulated TREX1 at the protein level (Figure 1D). We assessed the effects of RFWD3 on cellular exonuclease activity of TREX1, and found that knockdown of *RFWD3* reduced TREX1 exonuclease activity by ≈40% (Figure 1E), while overexpression of RFWD3 increased TREX1 activity by ≈50% (Figure 1F). Since TREX1 is able to degrade DNA inside cytosol,^[^ ^21^ ^]^ we assessed the levels of cytoplasmic dsDNA in *RFWD3* knockdown cells. Results showed that in A549 (Figure 1G,H), large cell lung cancer (LCLC) H1299 (Figure 1I,J), and LUAD H460 (Figure 1K,L) cells, transfected with negative control shRNA (shNC), cytoplasmic dsDNA was maintained at a relatively low level; in *RFWD3*‐knockdown cells, cytoplasmic dsDNA was significantly increased, and returned to a low level when RFWD3 was compensated by transfection of sh*RFWD3*‐resistant *RFWD3* (Figure 1G–L). These results indicated that RFWD3 has an important role in maintaining TREX1 functions, thus may play a role in inflammatory disorders and cancer immune escape.

### 2.2 RFWD3 Suppresses the cGAS‐STING Signaling Pathway via TREX1

**Figure 2:**
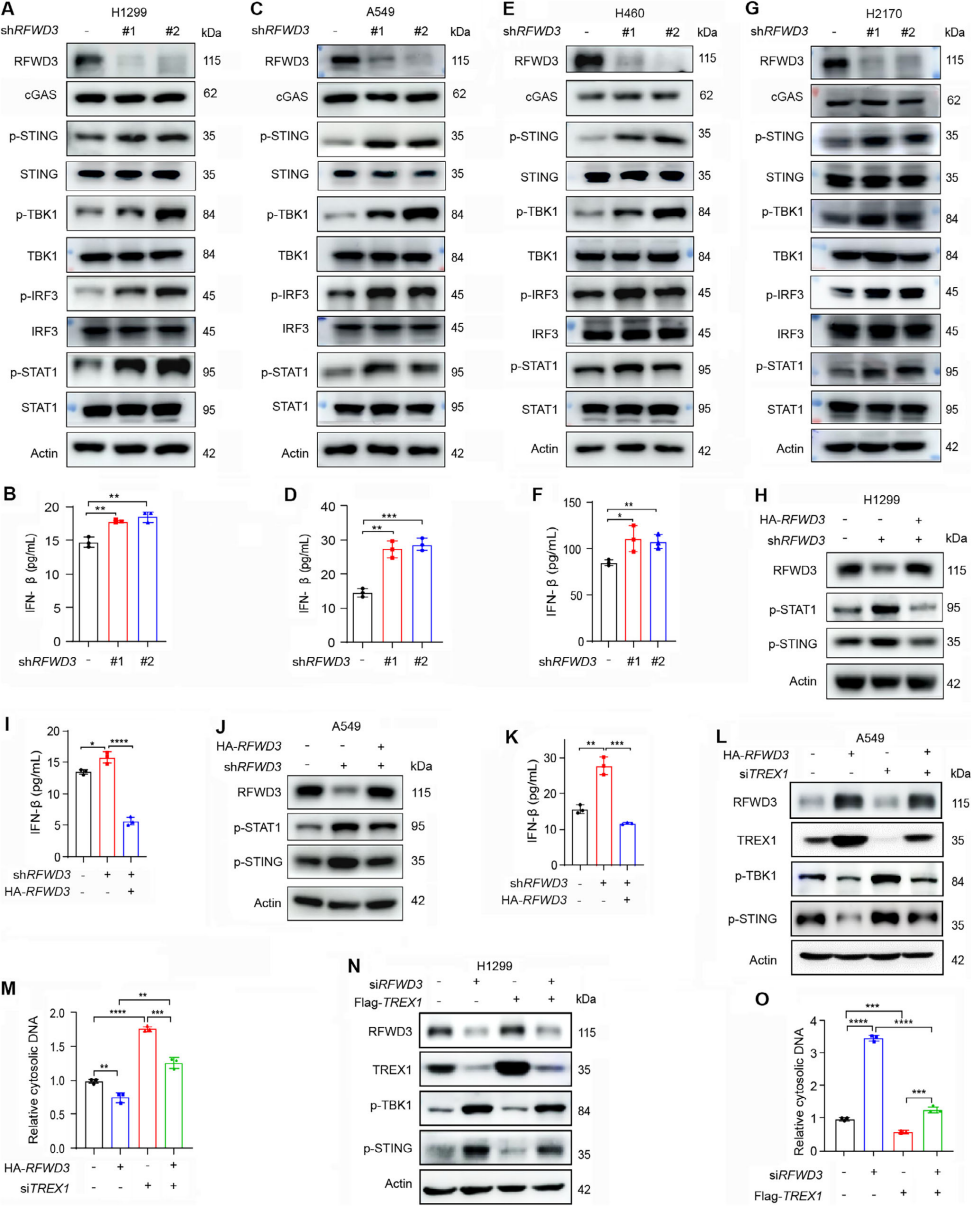
RFWD3 suppresses the cGAS‐STING signaling pathway via TREX1. A) H1299 cells were transfected with two different shRNAs against *RFWD3* and subjected to western blotting analysis using the indicated antibodies. B) IFN‐β was quantified using ELISA in the supernatant of H1299 cells transfected with two different shRNAs for *RFWD3*. C) A549 cells were transfected with two different shRNAs against *RFWD3*, and subjected to western blotting analysis using the indicated antibodies. D) IFN‐β was quantified using ELISA in the supernatant of A549 cells transfected with two different shRNAs for *RFWD3*. E) H460 cells were transfected with two different shRNAs against *RFWD3* and subjected to western blotting analysis using the indicated antibodies. F) IFN‐β was quantified using ELISA in the supernatant of H460 cells transfected with two different shRNAs for *RFWD3*. G) H2170 cells were transfected with shRNAs against *RFWD3* and subjected to western blotting analysis using the indicated antibodies. H) H1299 cells stably expressing *RFWD3* shRNA were transfected with HA‐*RFWD3* for 48 h. Immunoblotting was performed with the indicated antibodies. I) IFN‐β was quantified using ELISA in the supernatant of H1299 cells stably expressing *RFWD3* shRNA transfected with HA‐*RFWD3*. J) A549 cells stably expressing *RFWD3* shRNA were transfected with HA‐*RFWD3* for 48 h. Immunoblotting was performed with the indicated antibodies. K) IFN‐β was quantified using ELISA in the supernatant of A549 cells stably expressing *RFWD3* shRNA transfected with HA‐*RFWD3*. L) A549 cells transfected with si*TREX1* and HA‐*RFWD3* were subjected to western blotting using the indicated antibodies. M) Cytosolic DNA was isolated and quantified in A549 cells transfected with si*TREX1* and HA‐*RFWD3*. N) H1299 cells transfected with si*RFWD3* and Flag‐*TREX1* were subjected to western blotting using the indicated antibodies. O) Cytosolic DNA was isolated and quantified in H1299 cells transfected with si*RFWD3* and Flag‐*TREX1*. Error bars, SD. *p*‐values were calculated using a two‐tailed Student's *t*‐test. ^*^, *p*< 0.05; ^**^, *p*< 0.01; ^***^, *p*< 0.001; ^****^, *p*< 0.0001.

Cytosolic dsDNA is sensed by cGAS, subsequently activating STING signaling cascades and initiating IFN‐I production.^[^ ^22^ ^]^ We investigated whether RFWD3 regulates cGAS‐STING‐IFN signaling in human cancer cells, and found that knockdown of RFWD3 in H1299 cells (Figure S2A,B, Supporting Information) resulted in increased expression of phosphorylated‐STING (p‐STING), phosphorylated TANK‐binding kinase 1 (p‐TBK1), phosphorylated interferon regulatory factor 3 (p‐IRF3), and the IFN signal marker phosphorylated signal transducer and activator of transcription 1 (p‐STAT1),^[^ ^23^ ^]^ while the expression of cGAS, STING, TBK1, IRF3, and STAT1 was not significantly altered (**Figure** **2**A), though at mRNA level IRF3 and STAT1 was slightly upregulated (Figure S2C, Supporting Information). Knockdown of RFWD3 in H1299 cells increased IFN‐β concentration in supernatants (Figure 2B) and upregulated the expression levels of IFN‐β functional markers C‐X‐C motif chemokine ligand 10 (*CXCL10*), C‐C motif chemokine ligand 5 (*CCL5*), and Interferon‐stimulated gene (ISG) product 15 (*ISG15*)^[^ ^3^, ^24^ ^]^ (Figure S2D, Supporting Information). Knockdown of RFWD3 in A549 (Figure 2C,D) and H460 (Figure 2E,F) cells resulted in upregulation of p‐STING, p‐TBK1, p‐IRF3, p‐STAT1 (Figure 2C,E), and elevation of IFN‐β production (Figure 2D,F) and *CXCL10*, *CCL5*, *ISG15* expression levels (Figure S2D, Supporting Information). Silencing of RFWD3 in lung squamous cell carcinoma (LUSC) H2170 cells also led to upregulation of p‐STING, p‐TBK1, p‐IRF3, and p‐STAT1 (Figure 2G). Nevertheless, restoration of RFWD3 repressed the upregulated p‐STAT1 and p‐STING and reduced the increased IFN‐β production in both H1299 (Figure 2H,I) and A549 (Figure 2J,K) cells. Overexpression of RFWD3 did not perturb the ubiquitination of STING (Figure S2E, Supporting Information).

To determine the role of TREX1 on RFWD3‐induced inhibition of the STING pathway, cells were transfected with HA‐*RFWD3*/si*RFWD3* and/or si*TREX1*/Flag‐*TREX1*, and the downstream signaling molecules were evaluated. We found that while RFWD3 overexpression upregulated TREX1 and downregulated p‐TBK1 and p‐STING, knockdown of TREX1 reversed these effects (Figure 2L). Consistently, overexpression of RFWD3 reduced cytosolic DNA, while knockdown of TREX1 alleviated this phenomenon and increased cytosolic DNA in the cells (Figure 2M). On the contrary, silencing of RFWD3 caused downregulation of TREX1 and upregulation of p‐TBK1 and p‐STING (Figure 2N) and an increase in cytosolic DNA (Figure 2O), while overexpression of TREX1 reversed these effects (Figure 2N,O). These results suggested that TREX1 is required for the inhibitory effects of RFWD3 on the cGAS‐STING signaling pathway.

### 2.3 RFWD3 Binds and Protects TREX1 from TRIM24‐Mediated Proteasomal Degradation

**Figure 3:**
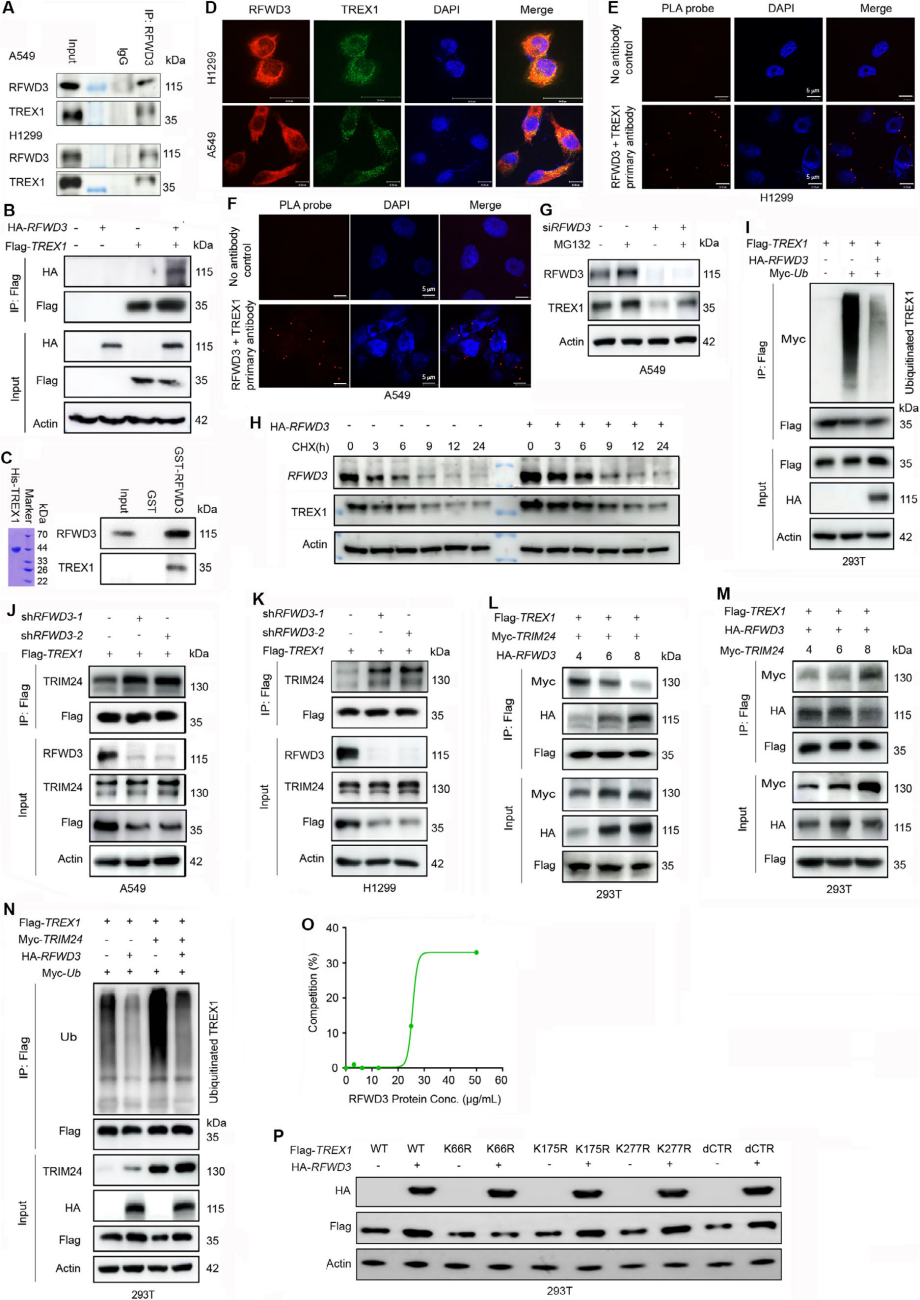
RFWD3 binds and protects TREX1 from TRIM24‐mediated proteasomal degradation. A) Co‐IP and immunoblotting assays using the indicated antibodies and cell lysates. B) HEK293T cells were transfected with HA‐*RFWD3* and Flag‐*TREX1* or with an empty vector, and Co‐IP and immunoblotting assays were performed using the indicated antibodies and cell lysates. C) GST pull‐down and western blot assays. Left, Coomassie blue staining of His‐TREX1 (1–314 aa). Right, GST or GST‐RFWD3 was incubated with His‐TREX1 in vitro, and the samples were analyzed using western blotting using antibodies against RFWD3 and TREX1. D) IF assays of H1299 and A549 cells using antibodies against RFWD3 (red), TREX1 (green), and DAPI to stain the nuclei (blue). Scale bars, 10 µm. E,F) Representative images of PLA using the Duolink in situ PLA Probe and anti‐RFWD3 and anti‐TREX1 antibodies in H1299 cells (E) and A549 cells (F). No primary antibodies were used as negative controls. Positive PLA signals indicating the RFWD3‐TREX1 complex are shown as red puncta. Scale bars, 10 µm. G) A549 cells were transfected with si*RFWD3*, treated with MG132 (10 mM) for 6 h, and subjected to western blotting. H) A549 cells were transfected with HA‐*RFWD3*, treated with CHX (20 mM) for different time points, and subjected to western blotting. I) HEK293T cells were co‐transfected with Flag‐*TREX1*, Myc‐ubiquitin (Ub), and HA‐*RFWD3* for 48 h. Co‐IP and immunoblotting were performed using the indicated antibodies and cell lysates. J) A549 cells stably expressing *RFWD3* shRNA were transfected with Flag‐*TREX1* for 48 h. Co‐IP and immunoblotting were performed using the indicated antibodies and cell lysates. K) H1299 cells stably expressing *RFWD3* shRNA were transfected with Flag‐*TREX1* for 48 h. Co‐IP and immunoblotting were performed using the indicated antibodies and cell lysates. L) HEK293T cells were co‐transfected with Flag‐*TREX1*, Myc‐*TRIM24*, and different amounts of HA‐*RFWD3* (4, 6, 8 µg) for 48 h. Co‐IP and immunoblotting assays using the indicated antibodies and cell lysates. M) HEK293T cells were co‐transfected with Flag‐*TREX1*, HA‐*RFWD3*, and different amounts of Myc‐*TRIM24* (4, 6, 8 µg) for 48 h. Co‐IP and immunoblotting assays using the indicated antibodies and cell lysates. N) HEK293T cells were co‐transfected with Flag‐*TREX1*, poly ubiquitin, Myc‐*TRIM24*, and HA‐*RFWD3* for 48 h. Co‐IP and immunoblotting were performed using the indicated antibodies and cell lysates. O) The TREX1‐binding capacity of TRIM24 incubated with different concentrations of RFWD3. The percentage of binding competition for RFWD3 was determined by ELISA. P) Flag‐*TREX1* or different Flag‐*TREX1* lysine mutants were transfected into HEK293T cells with or without Flag‐*RFWD3*. Cells were harvested, lysed, and subjected to western blotting.

We investigated how RFWD3 stabilizes TREX1. Co‐immunoprecipitation (co‐IP) analysis was performed using the endogenous proteins from A549 and H1299 cells to test the interaction between RFWD3 and TREX1, and results showed that RFWD3 was able to pull down TREX1 in both lines (**Figure** **3**A). In HEK293T cells transfected with HA‐*RFWD3* and Flag‐*TREX1*, TREX1 was able to pull down RFWD3 (Figure 3B). The GST pull‐down assay showed that GST‐RFWD3 could pull down His‐TREX1 in vitro (Figure 3C). To further confirm the interaction between these two proteins, we detected the co‐localization of RFWD3 and TREX1 in H1299 and A549 cells using immunofluorescence (IF) staining, and found that RFWD3 and TREX1 were mainly co‐localized in the cytoplasm (Figure 3D). The direct binding of RFWD3 and TREX1 was confirmed by the Duolink proximity ligation assay (PLA) that detects protein‐protein interactions in situ (at distances < 40 nm) at endogenous protein levels (Figure 3E,F). These results indicate that RFWD3 can bind TREX1.

To investigate how RFWD3 regulates TREX1 stability, we performed quantitative real‐time reverse transcription‐polymerase chain reaction (qPCR) analysis and found that si*RFWD3* did not affect TREX1 mRNA levels (Figure S3A, Supporting Information), suggesting that RFWD3 regulates TREX1 at the protein level. We found that treatment with the proteasome inhibitor MG132 (Figure 3G), but not autophagy inhibitor chloroquine (CQ; Figure S3B, Supporting Information), abolished TREX1 downregulation induced by si*RFWD3*, indicating that RFWD3 promotes TREX1 degradation via the ubiquitin‐proteasome pathway. Cycloheximide (CHX) chase assays showed that RFWD3 overexpression extended the half‐life of endogenous TREX1 from ≈4 h (control) to 10 h (Figure 3H; Figure S3C, Supporting Information). Consistent with this delayed degradation, the ubiquitination assay showed that RFWD3 markedly inhibited TREX1 ubiquitination in 293T cells (Figure 3I). However, RFWD3 did not affect TREX1 ubiquitination through K63‐linked chains (Figure S3D, Supporting Information).

TRIM24 is an E3 ubiquitin ligase for TREX1 ubiquitination and degradation.^[^ ^7^ ^]^ We evaluated the role of TRIM24 in RFWD3‐induced TREX1 upregulation, and found that knockdown (Figure S3E, Supporting Information) or overexpression (Figure S3F, Supporting Information) of RFWD3 did not affect the protein expression level of TRIM24. We speculated that RFWD3 may bind and sequestrate TREX1 from TRIM24 binding to evade ubiquitination and degradation. To test this possibility, a co‐IP assay was performed, and the results showed that the interaction between TREX1 and TRIM24 was significantly enhanced when RFWD3 was knocked down in A549 (Figure 3J) and H1299 cells (Figure 3K). Competitive co‐IP assay was performed to verify the competitive TREX1 binding mode between RFWD3 and TREX1, and results showed that the binding affinity of TRIM24 to TREX1 gradually decreased when RFWD3 was gradually increased (Figure 3L). Similarly, the binding affinity of RFWD3 to TREX1 gradually decreased when TRIM24 was gradually increased (Figure 3M). Furthermore, the ubiquitination assay showed that overexpression of TRIM24 promoted TREX1 ubiquitination, while RFWD3 significantly inhibited the TRIM24‐mediated TREX1 ubiquitination (Figure 3N). To further confirm that RFWD3 could competitively inhibit the TREX1‐TRIM24 interaction, a competition enzyme‐linked immunosorbent assay (ELISA) was performed, and the results showed that RFWD3 dose‐dependently inhibited the interaction between TREX1 and TRIM24 (Figure 3O).

Previous studies have shown that TRIM24 binds TREX1 at K66, K175, K277, and the C‐terminal region (CTR) and triggers its ubiquitination and subsequent degradation.^[^ ^7^ ^]^ We hypothesized that the site where RFWD3 competes with TRIM24 to bind to TREX1 may be one of these sites. To test this, mutations in these sites were introduced, and *TREX1* plasmids containing these mutations were transfected into 293T cells together with HA‐*RFWD3*. Results showed that while RFWD3 upregulated wild‐type TREX1, it failed to upregulate TREX1 containing the K66R mutation (Figure 3P), indicating that K66 could be the RFWD3‐binding site in TREX1 (Figure 3P). These findings indicated that RFWD3 enhances TREX1 stability by sequestrating it from TRIM24 to repress its ubiquitination and proteasomal degradation.

### 2.4 RFWD3 Reprograms Tumor Immune Microenvironment to Promote Carcinogenesis

**Figure 4:**
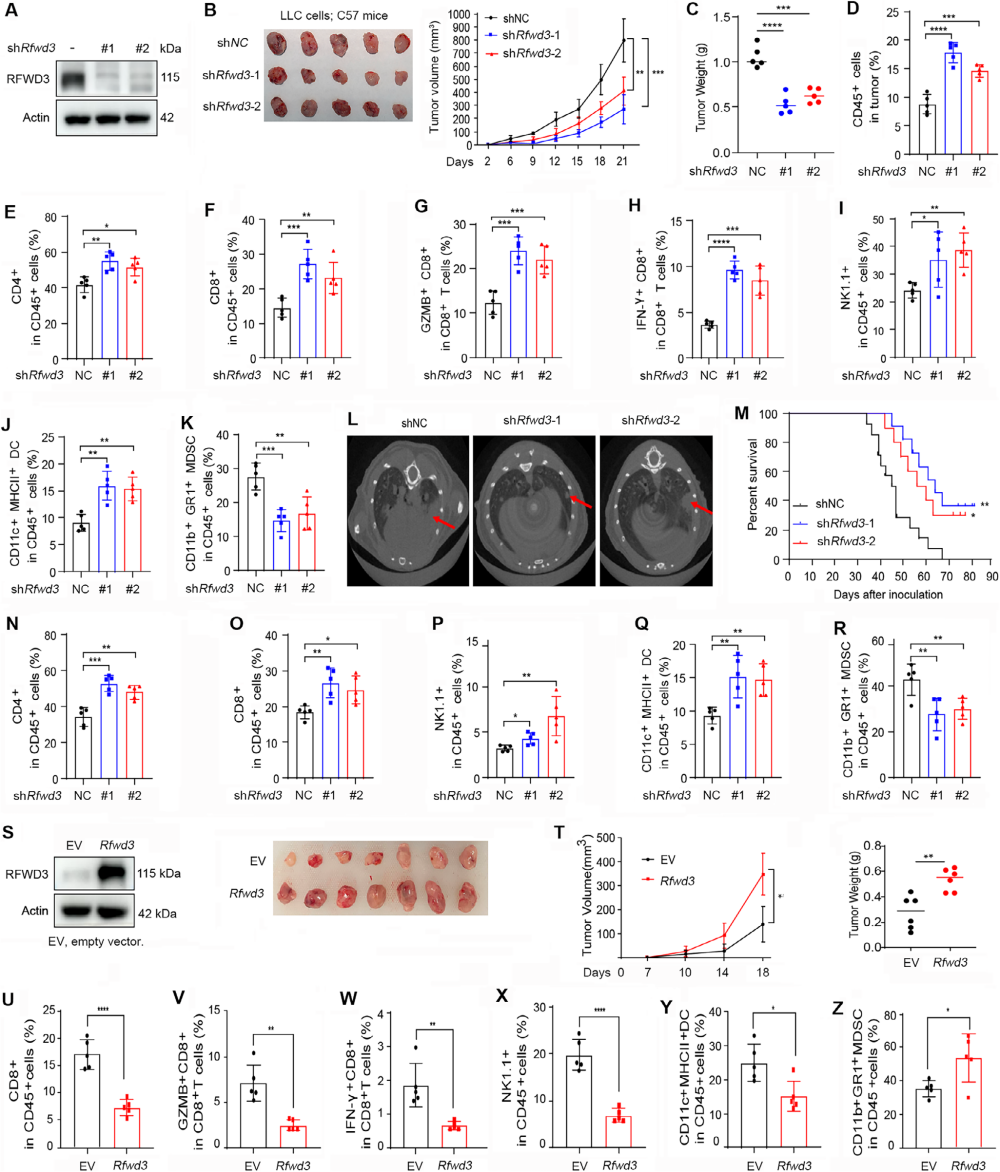
RFWD3 reprograms the tumor immune microenvironment to promote carcinogenesis. A) LLC cells were transfected with two different shRNAs against *Rfwd3* and subjected to western blotting analysis using the indicated antibodies. B,C) Tumor volume at the indicated days (B) and tumor weight at day 21 (C) after C57BL/6 mice were subcutaneously inoculated with LLC cells expressing control or *Rfwd3* shRNA. D–K) Flow cytometry of the percentages of the indicated types of tumor‐infiltrating immune cells in the tumor tissues taken from day 21 of C57BL/6 mice after inoculation with LLC cells expressing control or *Rfwd3* shRNA. L) LLC cells stably expressing control or *Rfwd3* shRNA were injected into the tail vein of C57BL/6 mice. Micro‐CT showing the tumor size in the mouse lung. The red arrow indicates a possible tumor. M) Kaplan‐Meier survival curves for C57BL/6 mice bearing LLC tumors treated as described in (L). N–R) Flow cytometry for the percentages of indicated types of tumor‐infiltrating immune cells in the tumor tissues taken from day 50 of C57BL/6 mice after tail vein inoculation with LLC cells stably expressing control or *Rfwd3* shRNA. S) LLC cells stably expressing *Rfwd3*, and subjected to western blotting using the indicated antibodies. C57BL/6 mice subcutaneously inoculated with LLC cells stably expressing control or RFWD3. Ex vivo images of the resected tumor. T) The tumor volume at the indicated days and tumor weight at day 18. U–Z) Flow cytometry for the percentages of indicated types of tumor‐infiltrating immune cells in the tumor tissues taken from day 18 of C57BL/6 mice after inoculation with LLC cells stably expressing control or RFWD3. Error bars, SD. *P*‐values were calculated using a two‐tailed Student's *t*‐test (C‐K, N‐R, T right ‐Z) or a Two‐way ANOVA (B, T left) test. Survival curves were calculated with the log‐rank test (M). ^*^, *p*< 0.05; ^**^, *p*< 0.01; ^***^, *p*< 0.001; ^****^, *p*< 0.0001.

We explored the role of RFWD3 in modulating the TREX1‐STING axis. First, C57BL/6 mice were subcutaneously transplanted with control or RFWD3 knockdown murine Lewis Lung Carcinoma (LLC) cells that are insensitive to immune checkpoint inhibitors (ICI),^[^ ^25^ ^]^ and we found that knockdown of *RFWD3* (**Figure** **4**A) significantly inhibited tumor growth (Figure 4B) and reduced tumor weight (Figure 4C). The impact of RFWD3 on the tumor immune microenvironment was analyzed by multicolor flow cytometry analysis (Figure S4A, Supporting Information). We found a significant increase in CD45^+^ immune cells in *Rfwd3*‐knockdown tumor samples compared with those in *Rfwd3*‐wild‐type tumors (Figure 4D). *Rfwd3* knockdown caused an increase in the number of tumor‐infiltrating CD4^+^ (Figure 4E) and CD8^+^ (Figure 4F) T cells. Among the CD8^+^ T cells, the Granzyme B‐ and IFN‐γ‐expressing populations that represent the activated cytotoxic T cells were increased in *Rfwd3*‐knockdown tumors (Figure 4G,H). We observed that *Rfwd3* knockdown in tumors increased the number of tumor‐infiltrating natural killer (NK) cells (Figure 4I) and dendritic cells (DC; Figure 4J) but decreased the number of myeloid‐derived suppressor cells (MDSC; Figure 4K).

We studied the effects of *Rfwd3* knockdown in tumor cells on the STING pathway in adjacent tumor‐infiltrating immune cells, by flow cytometric sorting of CD45^+^ cells from tumor tissues and western blot analysis of lysates of these CD45^+^ cells. We found that the expression levels of p‐STING, p‐TBK1, p‐IRF3, and p‐STAT1 were markedly increased in the *Rfwd3* knockdown group compared to the shNC group (Figure S4B, Supporting Information), indicating that knocking down *Rfwd3* in tumor cells results in activation of the STING pathway in adjacent immune cells.

We validated the effect of RFWD3 on TME in an orthotopic lung cancer model that was established by injection of shNC‐ or sh*Rfwd3*‐expressing LLC cells into C57BL/6 mice via the tail vein. We found that the lungs of mice that were injected with shNC‐treated LLC cells had larger tumors than those in mice injected with *Rfwd3* knockdown cells (Figure 4L). Overall survival of the *Rfwd3*‐knockdown group mice was significantly longer than that of the control group mice (Figure 4M). Compared with tumors in the control group mice, tumors of mice injected with sh*Rfwd3*‐expressing LLC cells had much more infiltrated CD4^+^ T, CD8^+^ T, NK cells, and DCs, and fewer MDSCs (Figure 4N–R).

On the contrary, RFWD3 overexpression promoted tumor growth and increased tumor weight (Figure 4S,T), inhibited the tumor‐infiltrating CD8^+^ T cells, Granzyme B‐ and IFN‐γ‐expressing CD8^+^ T cells (Figure 4U–W), and decreased the number of tumor‐infiltrating NK cells and DCs (Figure 4X,Y), but increased the proportion of MDSCs (Figure 4Z). We explored the effect of RFWD3 on the spatial distribution of immune cells by immunohistochemistry assay and HALO software analysis of CD8^+^ T cells in the tumor tissue of subcutaneous tumor‐bearing mice. We observed that *Rfwd3* knockdown led to enhanced infiltration of CD8^+^ T cells in the tumor, with a tendency for infiltration toward the interior of the tumor tissue (Figure S4C, Supporting Information). Thus, RFWD3 is able to reprogram TME to promote carcinogenesis, and inhibition of RFWD3 can effectively repress the progression of lung cancer in mice.

To determine whether the effects of RFWD3 on tumor growth are immune‐dependent or not, we subcutaneously inoculated shNC‐ or sh*Rfwd3*‐transfected LLC cells into NOD/ShiLtJGpt‐Prkdc^em26Cd52^Il2rg^em26Cd22^/Gpt (NSG) mice or Recombination‐Activating Gene 1 (*Rag1*) KO mice. We found that knockdown of *Rfwd3* did not significantly inhibit tumor growth in NSG models (Figure S5A–C, Supporting Information). By flow cytometry analyses, we found that the proportion of Ki67^+^ tumor cells and the mean fluorescence intensity (MFI) of Ki67 in tumors from NSG mice were not significantly altered (Figure S5D,E, Supporting Information). Consistently, knockdown of *Rfwd3* did not affect tumor growth (Figure S5F–H, Supporting Information) or Ki67^+^ cell proportion (Figure S5I, Supporting Information) in *Rag1* KO mice. These results indicated that the tumor‐promoting effects of RFWD3 mainly rely on the immune perturbation.

### 2.5 RFWD3 Shapes a Pro‐Tumor Microenvironment via TREX1‐STING‐IFN Axis

**Figure 5:**
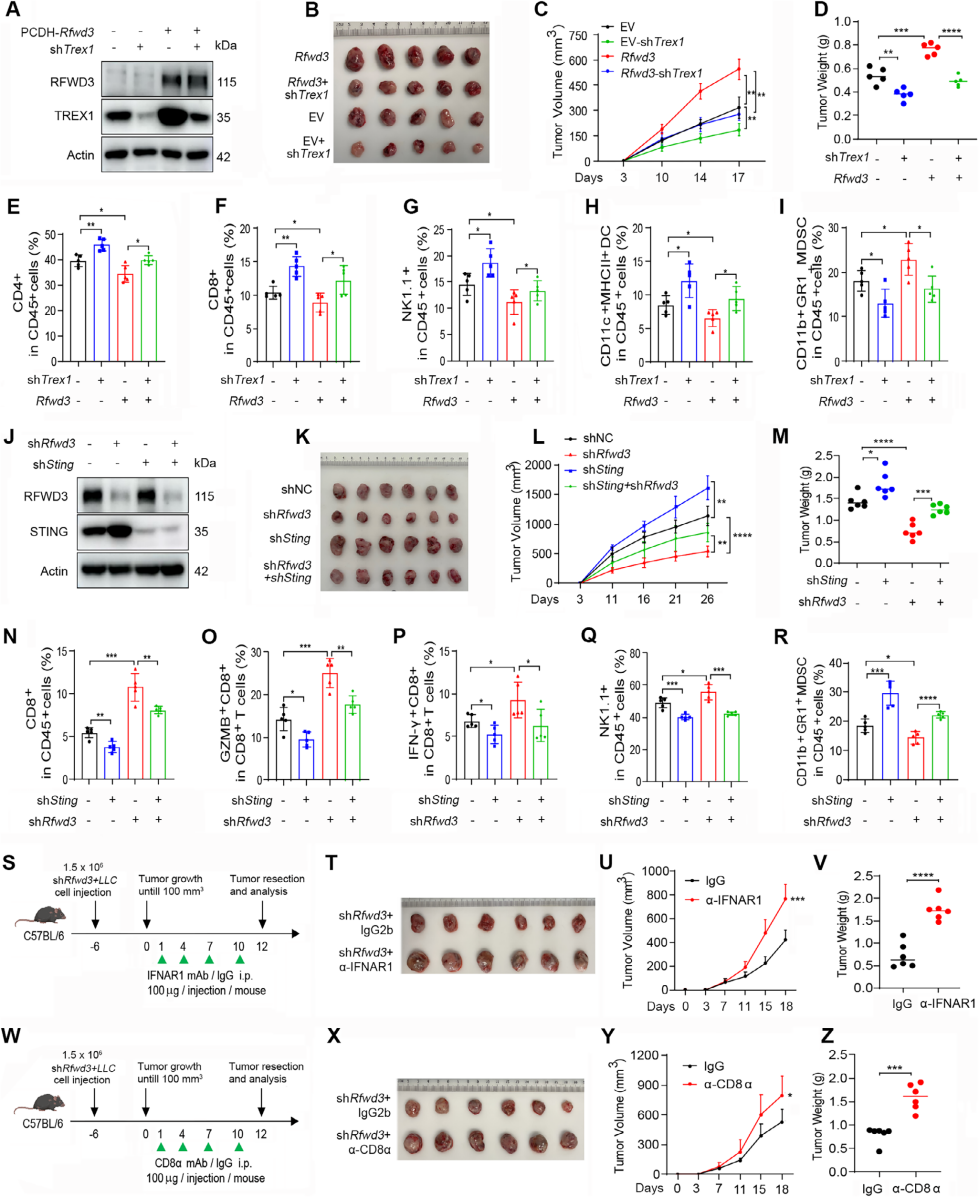
RFWD3 shapes a pro‐tumor microenvironment via the TREX1‐STING‐IFN axis. A) LLC cells stably expressing *Rfwd3* and *Trex1* shRNAs were subjected to western blotting using the indicated antibodies. B–D) C57BL/6 mice were subcutaneously inoculated with LLC cells stably expressing *Rfwd3* and *Trex1* shRNA. Ex vivo images of the resected tumors (B), tumor volume on the indicated days (C), and tumor weight on day 17 (D). E–I) Flow cytometry of the percentages of indicated types of tumor‐infiltrating immune cells in the tumor tissues taken from day 17 of C57BL/6 mice after inoculation with LLC cells stably expressing *Rfwd3* and *Trex1* shRNA. J) LLC cells stably expressing *Rfwd3* shRNA or *Sting* shRNA were subjected to western blotting using the indicated antibodies. K–M) C57BL/6 mice were subcutaneously inoculated with LLC cells stably expressing *Rfwd3* shRNA and *Sting* shRNA. Ex vivo images of the resected tumors (K), tumor volume on the indicated days (L), and tumor weight on day 26 (M). N–R) Flow cytometry of the percentages of indicated types of the tumor‐infiltrating immune cells in the tumor tissues taken from day 26 of C57BL/6 mice after inoculation with LLC cells stably expressing *Rfwd3* shRNA and *Sting* shRNA. S,T) Schematic of the experimental workflow. C57BL/6 mice subcutaneously inoculated with LLC cells stably expressing *Rfwd3* shRNA were injected with anti‐IFNAR antibody and/or IgG antibody as indicated (S). Ex vivo images of resected tumor (T). U,V) Tumor volume at indicated days (U) and tumor weight at day 18 (V). W,X) Schematic of the experimental workflow. C57BL/6 mice subcutaneously inoculated with LLC cells stably expressing *Rfwd3* shRNA were injected with anti‐CD8α antibody and/or IgG antibody as indicated (W). Ex vivo images of resected tumor (X). Y,Z) Tumor volume at indicated days (Y) and tumor weight at day 18 (Z). Error bars, SD. *p*‐values were calculated using a two‐tailed Student's *t*‐test (D‐I, M‐R, V, Z) or a Two‐way ANOVA test (C, L, U, Y). ^*^, *p*< 0.05; ^**^, *p*< 0.01; ^***^, *p*< 0.001; ^****^, *p*< 0.0001.

To investigate the role of TREX1 in RFWD3‐caused immune escape of cancer cells, LLC cells stably overexpressing Rfwd3 and/or silenced Trex1 were inoculated into C57 mice. We found that *Rfwd3* overexpression significantly promoted tumor growth, while *Trex1* knockdown significantly inhibited tumor growth, compared to control cell‐triggered tumors in the mice (**Figure** **5**A–D). However, *Rfwd3*‐promoted tumor growth was markedly inhibited by *Trex1* knockdown (Figure 5A–D), suggesting that RFWD3‐induced tumor progression was at least partially mediated by TREX1. Tumor‐infiltrating immune cells were dissociated from tumors and analyzed by flow cytometry, and we found that overexpression of *Rfwd3* significantly inhibited infiltration of CD4^+^ T cells, CD8^+^ T cells, NK cells, and DCs (Figure 5E–H), and increased MDSCs (Figure 5I). On the contrary, *Trex1* knockdown in the tumor increased tumor infiltration of CD4^+^ T cells, CD8^+^ T cells, NK cells, and DCs (Figure 5E–H), and decreased MDSCs (Figure 5I). However, *Rfwd3*‐triggered decrease in CD4^+^ T cells, CD8^+^ T cells, NK cells, and DCs, and increase in MDSCs were suppressed by *Trex1* knockdown (Figure 5E–I), suggesting that RFWD3‐induced pro‐tumor immune suppression depends on TREX1.

Genetic mutations in TREX1 are associated with autoimmune disease due to the inability of cells to eliminate cytosolic dsDNA.^[^ ^5^, ^26^ ^]^ This TREX1 deficiency‐related autoimmune response could be rescued by loss of STING,^[^ ^27^ ^]^ indicating that STING is required for this pathogenesis. Since RFWD3 negatively regulates the STING‐IFN pathway, we hypothesized that RFWD3 knockdown may facilitate the immunostimulatory effects on STING. To test this possibility, shNC, sh*Rfwd3*, sh*Sting*, and sh*Rfwd3*/sh*Sting*‐transfected LLC cells were subcutaneously injected into C57BL/6 mice, and tumor growth was monitored. Consistent with previous results, *Rfwd3* knockdown significantly inhibited tumor growth compared to the control group mice (Figure 5J–M). In contrast, *Sting* knockdown promoted tumor growth in comparison with the control group. Interestingly, the anti‐tumor effect of silencing Rfwd3 was markedly inhibited by *Sting* knockdown (Figure 5J–M), suggesting that the STING pathway has an important role in RFWD3‐induced tumorigenesis. Immunophenotyping analysis revealed that Rfwd3 knockdown increased the intra‐tumoral presence of CD8^+^ T cells, granzyme B^+^, IFN‐γ^+^, and NK cells, and decreased the intra‐tumoral presence of MDSCs. *Sting* knockdown reduced CD8^+^ T cells, granzyme B^+^, IFN‐γ^+^, and NK cells, and increased MDSCs in tumor tissues, and reversed the immunophenotype caused by *Rfwd3* knockdown (Figure 5N–R), suggesting the role of STING inhibition in RFWD3‐caused pro‐tumor TME.

IFN‐I signaling is a key effector of the STING pathway that promotes antigen presentation and DC activation.^[^ ^28^ ^]^ We assessed its role in RFWD3‐triggered TME abnormality by blocking IFN‐I signaling with a monoclonal antibody (mAb) against Interferon Alpha and Beta Receptor Subunit 1 (IFNAR1; Figure 5S). We found that compared with IgG‐treated tumors, treatment with the anti‐IFNAR1 antibody (α‐IFNAR1) significantly promoted the growth of *Rfwd3* knockdown cells in vivo (Figure 5T–V) without suppression of body weight of the mice (Figure S6A, Supporting Information). Anti‐IFNAR1 treatment significantly decreased the infiltration of CD4^+^ T cells, CD8^+^ T cells, NK cells, and DCs, and increased the proportion of MDSCs (Figure S6B–F, Supporting Information). Similarly, anti‐CD8α mAb (α‐CD8α) treatment significantly promoted the growth of *Rfwd3* knockdown cells in vivo (Figure 5W–Z) without suppression of body weight of the mice (Figure S6G, Supporting Information). Anti‐CD8α treatment significantly decreased the infiltration of CD4^+^ T cells, CD8^+^ T cells, NK cells, and DCs, and increased the proportion of MDSCs (Figure S6H–L, Supporting Information). These results indicated that suppression of IFN signaling underlies RFWD3‐induced pro‐tumor TME.

To investigate whether the conditioned media from *Rfwd3*‐knockdown tumor cells can activate immune cells, we co‐cultured LLC cells and RAW‐Lucia ISG cells that were generated from the murine RAW 264.7 macrophage cell line by stable integration of an interferon regulatory factor (IRF)‐inducible Lucia luciferase reporter construct. By flow cytometry, we analyzed the expression of cluster of differentiation 80 (CD80) on RAW cells and found that the CD80 expression level was upregulated in the *Rfwd3* knockdown group (Figure S7A, Supporting Information), indicating that knocking down *Rfwd3* upregulates costimulatory molecule expression and can activate RAW cells. Meanwhile, we co‐cultured LLC cells, bone marrow‐derived dendritic cells (BMDCs), and CD8^+^ OT1 T cells in the presence of OVA protein, and collected the culture supernatants to detect the level of cytokines. Results showed that the tumor necrosis factor‐alpha (TNF‐α) secreted by T cells in the *Rfwd3* knockdown group was higher than control group (Figure S7B, Supporting Information), indicating that knocking down RFWD3 can increase the antigen‐presenting function of antigen‐presenting cells (APC) and promote T cell function.

### 2.6 Inhibition of RFWD3 Sensitizes Unresponsive Tumor Cells to PD‐L1 Inhibition

**Figure 6:**
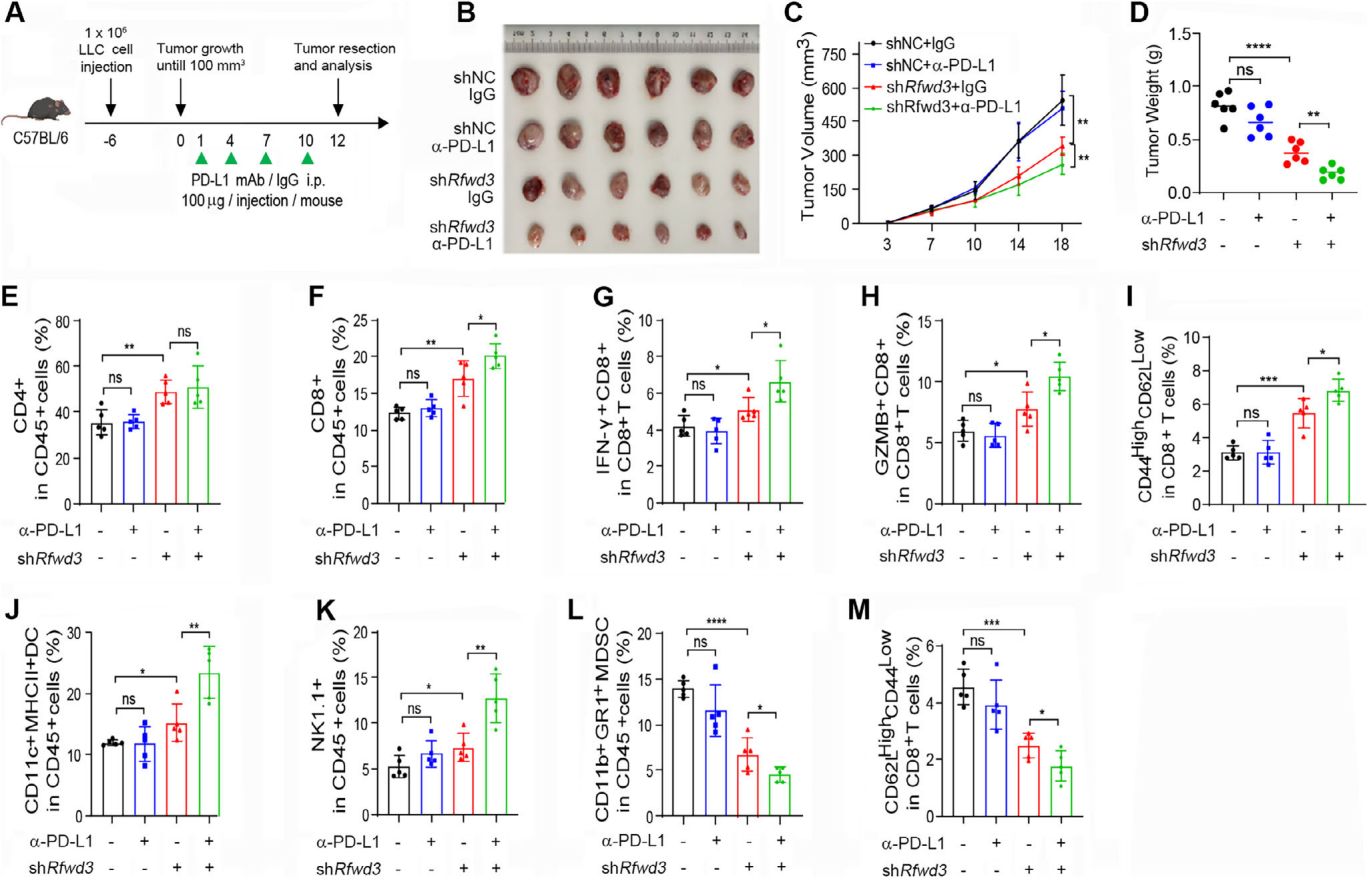
Inhibition of RFWD3 sensitizes unresponsive tumor cells to PD‐L1 inhibition. A,B) Schematic of the experimental workflow. C57BL/6 mice subcutaneously inoculated with LLC cells stably expressing shNC or *Rfwd3* shRNA were injected with anti‐PD‐L1 and/or anti‐IgG antibodies, as indicated (A). Ex vivo images of the resected tumor (B). C,D) Tumor volume on the indicated days (C) and tumor weight on day 18 (D). E–M) Flow cytometry of the percentages of indicated types of tumor‐infiltrating immune cells in the tumor tissues taken from day 18 of C57BL/6 mice subcutaneously inoculated with LLC cells and treated as described in (A). Error bars, SD. *P*‐values were calculated using a two‐tailed Student's *t*‐test (D–M) or a Two‐way ANOVA test (C). ^*^, *p*< 0.05; ^**^, *p*< 0.01; ^***^, *p*< 0.001; ^****^, *p*< 0.0001; ns, not significant.

Inhibition of RFWD3 activates the IFN pathway to enhance the innate immune response, suggesting that targeting RFWD3 could convert immunosuppressive “cold” tumors into immunoreactive “hot” tumors to improve the tumor response to ICI therapy. To test this hypothesis, we investigated the combined effects of RFWD3 inhibition (by sh*Rfwd3* transfection) and an anti‐PD‐L1 antibody (α‐PD‐L1) in LLC tumor‐bearing mice (**Figure** **6**A). We found that at 100 µg dosage, α‐PD‐L1 did not significantly inhibit LLC tumor growth (Figure 6B–D), consistent with previous reports.^[^ ^25^ ^]^ Knockdown of *Rfwd3* inhibited tumor growth and significantly improved the therapeutic efficacy of α‐PD‐L1 (Figure 6B–D). We found that at 100 µg dosage, α‐PD‐L1 did not significantly perturb immune cells, but significantly enhanced sh*RFWD3*‐induced increase in CD4^+^ T cells, CD8^+^ T cells, IFN‐γ^+^, Granzyme B^+^, and CD44^high^CD62L^low^ (memory T cells) CD8^+^ T cells, DCs, NK cells, and further reduced MDSCs and CD62L^high^CD44^low^ Naïve T cells (Figure 6E–M). These results indicate that RFWD3 inhibition enhanced the anti‐tumor effects of the PD‐L1 antibody by shaping an inflamed TME.

To further validate the role of the STING pathway in the synergistic effect between RFWD3 inhibition and PD‐L1 blockade, we divided C57BL/6 mice into two groups after subcutaneously inoculating LLC‐*Rfwd3* cells, one receiving PD‐L1 treatment and the other receiving PD‐L1 combined with STING pathway inhibitor C‐176 (Figure S8A, Supporting Information). Results showed that the tumor growth inhibition effect induced by *Rfwd3* knockdown combined with PD‐L1 was reversed after blocking the STING pathway (Figure S8B–D, Supporting Information), indicating that the synergistic effect between RFWD3 inhibition and PD‐L1 blockade depends on STING pathways.

### 2.7 RFWD3 Expression Level is Inversely Associated with Overall Survival of NSCLCs

**Figure 7:**
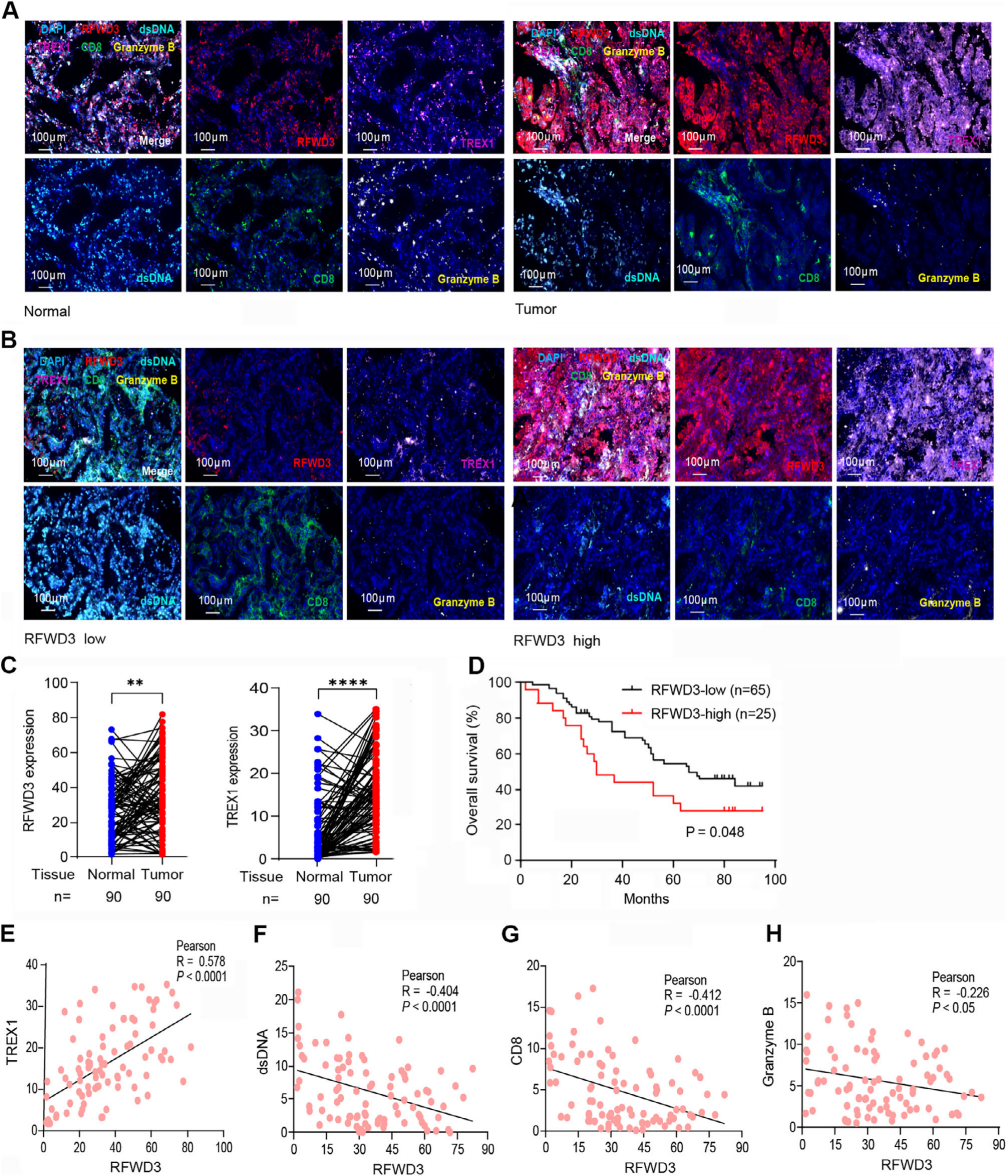
RFWD3 expression level is inversely associated with the overall survival of patients with NSCLC. A) Representative mIHC images of adjacent normal and tumor tissues from NSCLC patients. Scale bars = 100 µm. B) Representative mIHC images of NSCLC patients in RFWD3‐high and RFWD3‐low groups. Scale bars = 100 µm. C) The expression levels of *RFWD3* and *TREX1* in the adjacent normal and tumor tissues from NSCLC patients. D) The Kaplan–Meier survival curve of patient OS corresponding to the optimum density cutoff of RFWD3. E–H) The correlation between RFWD3 and TREX1 (E), dsDNA (F), CD8 (G), and Granzyme B (H) in NSCLC patients was analyzed. Error bars, SD. *P*‐values were calculated using a two‐tailed Student's *t*‐test (C). ^**^, *p*< 0.01; ^****^, *p*< 0.0001. Correlations were assessed using Pearson's correlation coefficient (R; E‐H). Survival curves were calculated with the log‐rank test (D).

We tested the expression of RFWD3, TREX1, dsDNA, Granzyme B, and CD8 in 90 patients with treatment‐naïve LUAD (**Table** **1**) by multiplex immunohistochemistry (mIHC) staining. We found that RFWD3 and TREX1 were highly expressed in tumor tissues when compared with the counterpart adjacent normal lung tissues (**Figure** **7**A). The expression level of RFWD3 was low in some patients and high in others (Figure 7B,C). The optimum density cutoff value of RFWD3 was determined using X‐tile software, and RFWD3‐high was associated with poor overall survival (OS) of the patients (Figure 7D). By analyzing the mIHC results of RFWD3‐high and RFWD3‐low groups, we found that RFWD3 expression level was positively correlated with TREX1 level (Figure 7E), but negatively correlated with dsDNA (Figure 7F), CD8 (Figure 7G), and Granzyme B (Figure 7H) expression levels.

**Table 1:** Baseline characteristics of the 90 patients with NSCLC.

Characteristics	Total	RFWD3‐Low, n [%]	RFWD3‐High, n [%]	*p* Value*
n	90	65 (72.2)	25 (27.8)	
Age				
≤ 60	45	34 (75.6)	11 (24.4)	0.64
>60	45	31 (68.9)	14 (31.1)	
Sex				
Male	49	35 (71.4)	14 (28.6)	0.99
Female	41	30 (73.2)	11 (26.8)	
Greatest tumor diameter				
< 4	57	43 (75.4)	14 (24.6)	0.47
≥ 4	33	22 (66.7)	11 (33.3)	
Histologic grade				
≤ II	64	48 (75)	16 (25)	0.44
> II	26	17 (65.4)	9 (34.6)	
Stage				
I‐II	69	51 (53.9)	18 (26.1)	0.58
III‐IV	21	14 (66.7)	7 (33.3)	

*P* Value, the Fisher's exact test.

### 2.8 RFWD3 is Upregulated by Exposure to Air Pollutants

**Figure 8:**
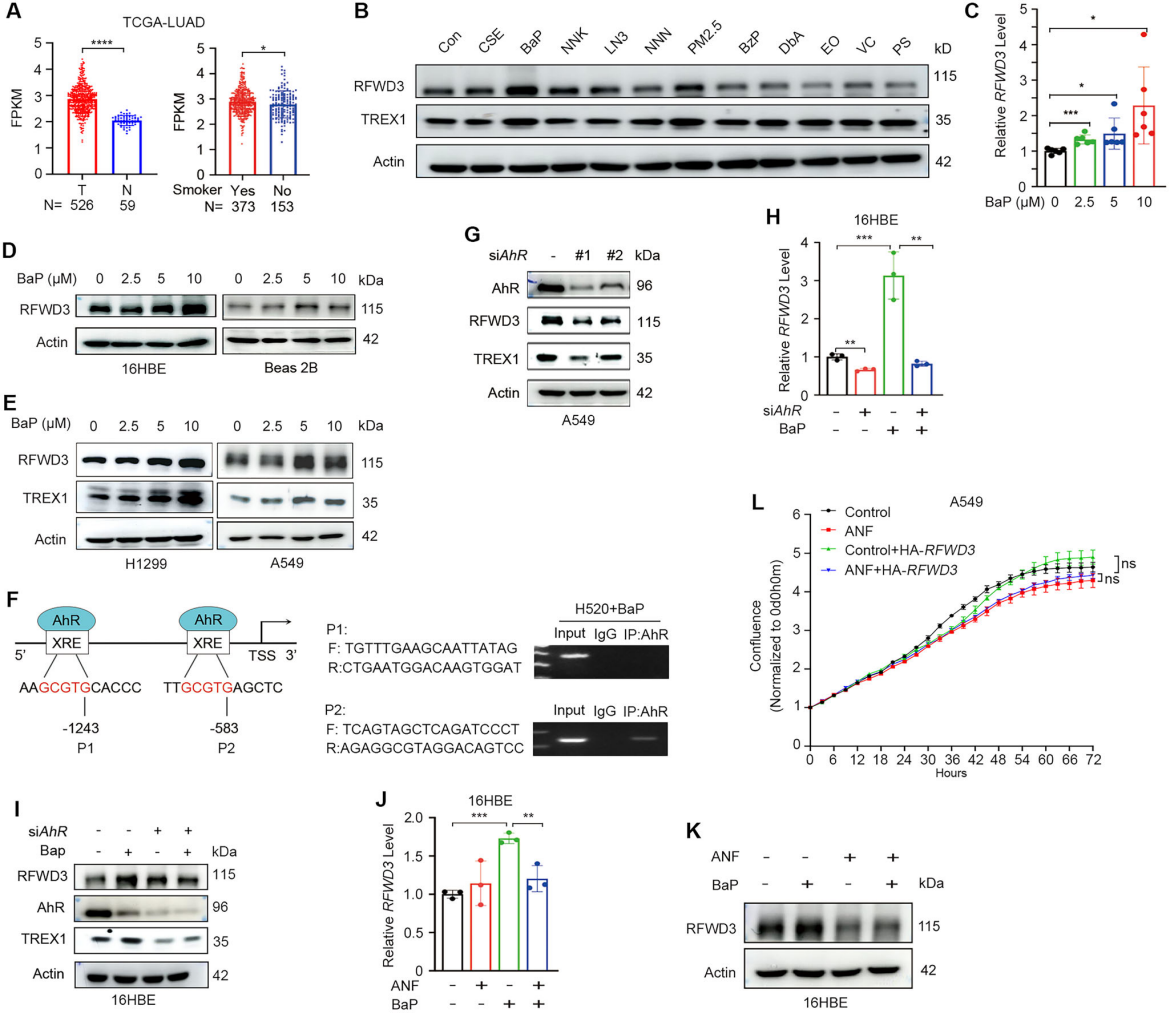
Environmental pollutants induce upregulation of RFWD3. A) The expression level of RFWD3 in tumor tissues and normal lung tissues of patients with LUAD (left), and in patients with NSCLC who were smokers or non‐smokers (right). Data were downloaded from the TCGA database. FPKM, Fragments Per Kilobase of exon model per Million mapped fragments. B) Effects of tobacco and PM_2.5_ carcinogens on the expression level of RFWD3 in 16HBE cells. The cells were treated with cigarette smoke extract (CSE; 10%), PM_2.5_ (10 µg mL^−1^), or indicated compounds at 10 µM for 48 h, lysed, and subjected to western blotting assays using indicated antibodies. PM_2.5_, particulate matter of smaller than 2.5 µm in diameter; BaP: benzo(a)pyrene; NNK: nicotine‐derived nitrosaminoketone; LN3: nicotine; NNN: N’‐nitrosonornicotine; BzP: benzo(g,h,i)perylene; DbA: dibenzo(a,h)anthracene; EO: ethylene oxide; VC: vinyl chloride; PS: polystyrene. C,D) 16HBE cells were treated with indicated concentrations of BaP for 48 h, lysed, and subjected to qPCR (C) or western blotting (D) to detect the expression of RFWD3 at mRNA and protein levels. E) H1299 and A549 cells were treated with indicated concentrations of BaP for 48 h, lysed, and subjected to western blotting using indicated antibodies. F) The potential AhR‐binding sites in the *RFWD3* promoter (left). ChIP‐seq experiments were performed, and results showed that P2 is the AhR‐binding site (right). TSS, transcription start site; XRE, xenobiotic‐responsive element. G) Knockdown of AhR by siRNA led to downregulation of RFWD3 and TREX1 in A549 cells. H,I) 16HBE cells were transfected with si*AhR*, treated with BaP at 10 µM for 48 h, lysed, and subjected to qPCR (H) or western blotting (I) to detect the expression of RFWD3. J,K) 16HBE cells were treated with BaP and/or ANF at 10 µM for 48 h, lysed, and subjected to qPCR (J) or western blotting (K) to detect the expression of RFWD3. L) A549 cells were treated with ANF at 10 µM or PBS for 48 h, transfected with HA‐*RFWD3*, and monitored by Incucyte for cell proliferation. Error bars, SD. *p*‐values were calculated using a two‐tailed Student's *t*‐test. ^*^, *p*< 0.05; ^**^, *p*< 0.01; ^***^, *p*< 0.001; ns, not significant.

More than 90% of lung cancers are associated with exposure tosmohaze,^[^ ^29^ ^]^ and our previous work showed that RFWD3 expression is significantly elevated in smokers compared with nonsmokers.^[^ ^20^ ^]^ We analyzed TCGA datasets^[^ ^30^ ^]^ (https://cghub.ucsc.edu/, with approval by the National Institutes of Health number #24437‐4) and confirmed that smoker patients expressed a higher level of RFWD3 than nonsmoker patients (**Figure** **8**A). Therefore, we tested the effects of smohaze on the expression of RFWD3. Results showed that treatment of the human normal lung epithelial 16HBE cells with cigarette smoke extract (CSE; 10%) or particulate matter of smaller than 2.5 µm in diameter (PM_2.5_^[^ ^31^ ^]^; 10 µg mL^−1^) for 48 h resulted in upregulation of RFWD3 expression at the protein level (Figure 8B). We tested the impact of 9 common air pollutants, and found that treatment with benzo(a)pyrene (BaP), Benzo(g,h,i)perylene (BzP), nicotine (LN3), and nicotine‐derived nitrosaminoketone (NNK) at 10 µM for 48 h induced upregulation of RFWD3, with BaP as the most significant one (Figure 8B). We showed that BaP upregulated RFWD3 at both the mRNA and protein levels in a dose‐dependent manner (Figure 8C,D). BaP also upregulated the expression of RFWD3 and TREX1 in NSCLC H1299 and A549 cells (Figure 8E).

AhR is an environmental sensor that can be activated by agonists (such as BaP) and plays a critical role in environmental carcinogen‐induced tumorigenesis.^[^ ^29^, ^32^ ^]^ When exposed to polycyclic aromatic hydrocarbons (PAHs) such as BaP, cytoplasmic AhR binds the ligand and enters the nucleus, forming heterodimers with AhR nuclear translocator (ARNT). The AhR/ARNT heterodimer binds the xenobiotic‐responsive element (XRE) and induces the transcription of target genes.^[^ ^33^ ^]^ We found two XRE‐like sequences, 5′‐ AAGCGTGCACCC ‐3′ and 5′‐ TTGCGTGAGCTC ‐3′, that are located at −1243 bp (P1 site) and −538 bp (P2 site) upstream of the transcription start site (TSS) (Figure 8F). By chromatin‐immunoprecipitation (ChIP)‐PCR assays, we found that the P2 site represented an AhR‐binding site in *the RFWD3* promoter region (Figure 8F).

Silencing of AhR by siRNA suppressed the expression of RFWD3 and TREX1 (Figure 8G). When AhR was knocked down by si*AhR*, BaP‐induced upregulation of RFWD3 at both mRNA (Figure 8H) and protein (Figure 8I) levels was markedly inhibited in 16HBE cells. Treatment of the cells with AhR inhibitor Alpha‐Naphthoflavone (ANF) inhibited BaP‐induced upregulation of RFWD3 at mRNA (Figure 8J) and protein (Figure 8K) levels. Meanwhile, IncuCyte detection showed that both ANF and RFWD3 had no significant effect on tumor cell proliferation (Figure 8L). The above results indicate that air pollutants can upregulate the expression of RFWD3 through AhR transcription.

## 3 Discussion

The tobacco epidemic is one of the biggest public health threats the world has ever faced, killing over 8 million people a year around the world.^[^ ^34^ ^]^ Meanwhile, 99% of the world's population was living in places where the World Health Organization (WHO) air quality guidelines were not met.^[^ ^35^ ^]^ More than 90% of NSCLCs are associated with tobacco smoke or exposure to household or ambient air pollution.^[^ ^29^ ^]^ Tobacco smoke and PM_2.5_ cause a large number of mutations in the genome, abnormalities in epigenomics, and initiation of tumor‐promoting chronic inflammation.^[^ ^29^, ^36^ ^]^ Environmental carcinogens facilitate immune escape by inducing expression of PD‐L1,^[^ ^37^ ^]^ PD‐L2,^[^ ^38^ ^]^ and Indoleamine 2,3‐dioxygenase 1.^[^ ^39^ ^]^ Here, we showed that tobacco smoke, PM_2.5_, and component BaP upregulated RFWD3 in an AhR‐dependent manner, and RFWD3 represented an AhR target gene. RFWD3 inhibited cGAS‐STING signaling by stabilizing TREX1 and degrading dsDNA, leading to the accumulation of MDSCs and suppression of anti‐tumor immunity. Hence, environmental carcinogens may cause tumorigenesis through complicated mechanisms, and environmental exposures may influence how patients respond to therapy. Identification of key molecules in this process is critical to the development of preventive and therapeutic approaches for this disease.

In cancer cells, cytosolic DNA can originate from genomic instability, mitochondrial DNA, micronuclei rupture, and cell death processes.^[^ ^40^ ^]^ To avoid activation of the SITNG‐IFN cascade, cancer cells use TREX1 to degrade abnormally accumulated cytosolic DNA, and TREX1 can degrade dsDNA, single‐stranded DNA (ssDNA), RNA:DNA hybrids, and some RNA species.^[^ ^41^, ^42^ ^]^ While previous studies^[^ ^7^ ^]^ have shown that TRIM24, a p53‐inducible E3 ligase, targets TREX1 for degradation to activate the cGAS‐STING pathway, our data reveal an opposing mechanism whereby RFWD3, as a true TREX1 interactor, protects it from degradation, thereby suppressing STING activation. Importantly, RFWD3 is upregulated by AhR in response to environmental carcinogens, providing a p53‐independent means of TREX1 stabilization and immune evasion in NSCLC. These results provided an explanation for TREX1 overexpression in cancer cells, in the presence of its degrader, TRIM24 expression. These data also suggested that an E3 ligase is able to protect a substrate protein, confirmed by a previous report that the E3 ubiquitin ligase TRIM26 binds to the N‐terminal region of the transcription factor SOX2 and protects it from WWP2‐mediated polyubiquitination and proteasomal degradation.^[^ ^43^ ^]^

The cGAS/STING signaling has been shown to exert tumor‐suppressing effects via production of IFN‐I, promotion of cellular senescence through senescence‐associated secretory phenotype (SASP), and activation of antigen‐presenting cells. Loss‐of‐function has been seen in STING and cGAS, and cancer may hijack epigenetic inactivation of cGAS/STING signaling to evade immune surveillance in facilitating tumorigenesis.^[^ ^3^, ^44^ ^]^ Hence, pharmacological activation of this signaling has been proposed to be a promising anti‐cancer strategy. Dozens of STING agonists, as well as cGAS inhibitors and TBK1 inhibitors, have been developed, and STING reactivation can restore IFN signal activation and resensitize tumors to ICI.^[^ ^45^ ^]^ However, the clinical therapeutic efficacies of STING agonists in cancer patients remain unsatisfactory so far.^[^ ^44^ ^]^ Furthermore, recent studies show that cGAS‐STING also bears pro‐tumor functions, e.g., inducing pro‐tumor chronic inflammation^[^ ^46^ ^]^ and activation of NF‐κB^[^ ^47^ ^]^ and STAT1 signaling through production of TNF‐α and IFN‐α.^[^ ^48^ ^]^ These results suggest the dark side of STING agonists in treating cancer.

Other cGAS‐STING pathway molecules have been considered to be therapeutic targets for cancer treatment. Since TREX1 is overexpressed in many types of cancer to limit the activation of STING‐IFN signaling,^[^ ^49^ ^]^ it triggers an invasive phenotype in cancer cells,^[^ ^50^ ^]^ and reduces the anti‐tumor immune response to cytotoxic T lymphocyte‐associated antigen‐4 (CTLA‐4) and PD‐1 therapy,^[^ ^51^, ^52^ ^]^ it is regarded as a target for STING activation.^[^ ^7^ ^]^ Indeed, TREX1 inactivation unleashes cancer cell STING‐IFN signaling,^[^ ^53^ ^]^ amplifies cancer cell immunogenicity, recruits immune effector cells, primes NK cell‐mediated killing, enhances anti‐tumor efficacy of ICI,^[^ ^54^ ^]^ and overcomes ICI resistance.^[^ ^53^ ^]^ Currently, several TREX1‐targeting small molecules are being tested in preclinical models.^[^ ^55^, ^56^ ^]^ However, mutations of TREX1 cause a series of autoimmune diseases, including Aicardi–Goutières syndrome (AGS), systemic lupus erythematosus (SLE), and familial chilblain lupus (FCL),^[^ ^57^, ^58^ ^]^ suggesting the risk concerns of TREX1‐targeting agents. Furthermore, we found that the expression level of TREX1 was not associated with the overall survival of NSCLC patients. In addition, increased methylation and epigenetic silencing of TREX1 are observed in patients with small cell lung cancer, and are associated with increased sensitivity of tumor cells to anti‐mitotic drugs, ATR, and CDK inhibitors.^[^ ^10^ ^]^ Hence, the potential of TREX1 inhibitors remains to be determined.

**Figure 9:**
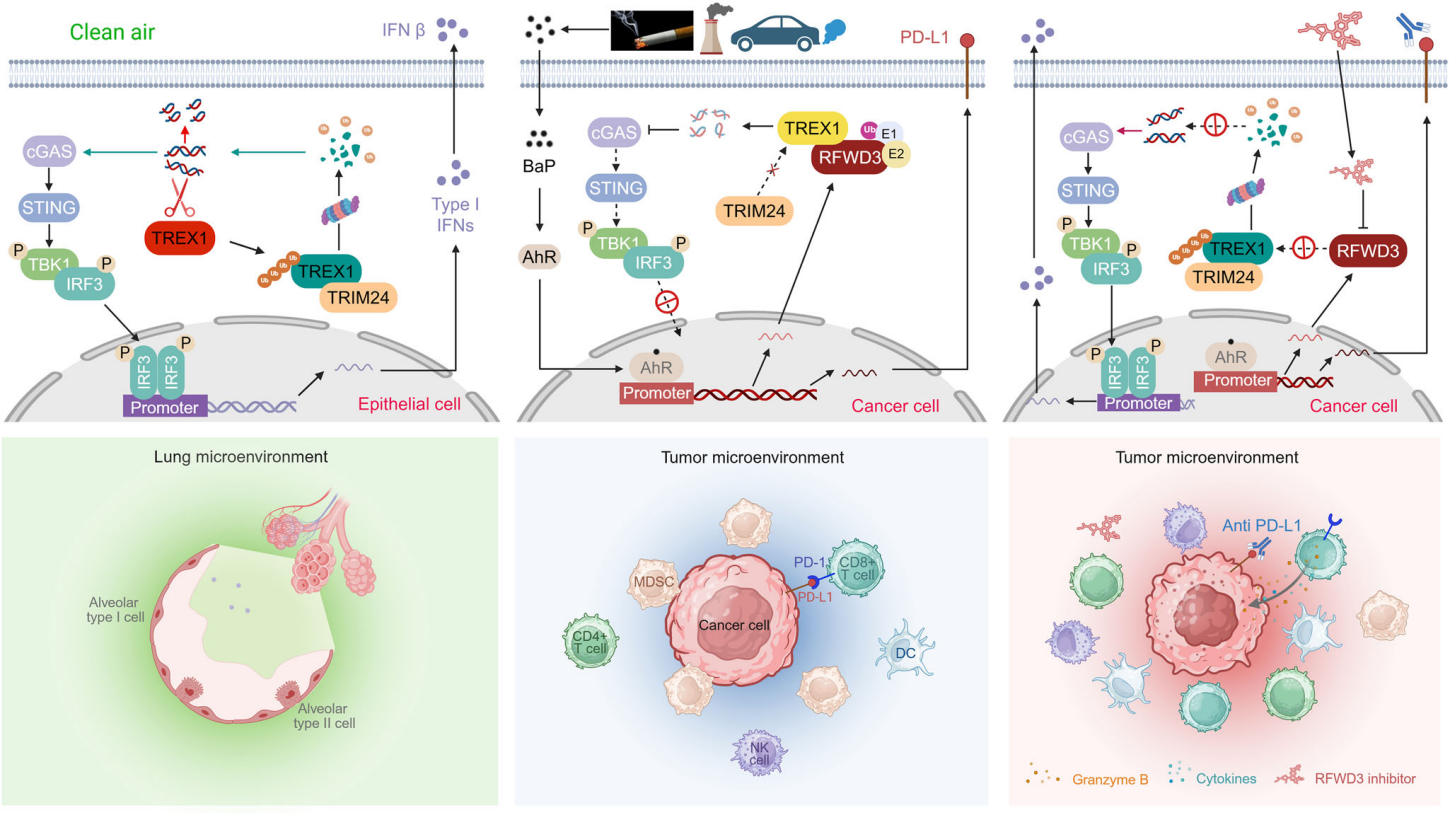
Schematic illustration of RFWD3‐mediated pro‐tumor immunity. (Left) In lung epithelial cells exposed to clean air, TREX1 undergoes TRIM24‐mediated proteasomal degradation, leading to accumulation of dsDNA, activation of cGAS‐STING signaling, and production of IFN‐I, such as IFNβ. (Middle) Tobacco and PM_2.5_ induce upregulation of RFWD3 via the BaP‐AhR pathway, resulting in stabilization of TREX1, inactivation of cGAS‐STING signaling, upregulation of PD‐L1,^[^ ^37^ ^]^ and an immunosuppressive tumor microenvironment. (Right) Inhibition of RFWD3 restores TREX1 degradation, dsDNA accumulation, cGAS‐STING activation, and IFN production. Combined use of RFWD3 inhibition and ICI exerts synergistic therapeutic efficacy. Created with BioRender.com.

Over the past two decades, there has been enormous progress in the understanding of the biology and management of NSCLC, with targeted therapies and ICIs providing significant improvement in survival. However, disease progression remains inevitable for most patients with advanced NSCLC, and there will be an estimated 1.5 million NSCLC deaths globally in 2022.^[^ ^59^ ^]^ Hence, novel therapeutic targets and prognostic biomarkers are still desired to tame NSCLC. RFWD3 is an essential component of the cellular machinery that safeguards DNA integrity. Recently, studies have demonstrated the emerging roles of RFWD3 in cancers. RFWD3 is overexpressed and inversely associated with the overall survival of several types of cancers.^[^ ^17^, ^18^, ^19^, ^20^, ^60^ ^]^ We found that the RFWD3 expression level was inversely associated with the overall survival of patients with NSCLCs. RFWD3 expression was positively associated with TREX1 but inversely associated with the expression level of dsDNA, CD8, and Granzyme B; hence, high RFWD3 may mark immune “cold” tumors, and RFWD3 levels could be used as a biomarker for immunotherapy response. Knockdown of RFWD3 inhibited proliferation and increased secretion of IFN‐β at the cellular level, and repressed tumor growth with an increase in CD4^+^, CD8^+^, Granzyme B^+^/CD8^+^, IFN‐γ/CD8^+^, NK, and DC cells and a decrease in MDSCs. RFWD3 inhibition enhanced the anti‐tumor efficacy of ICI in vivo (**Figure** **9**). Inhibition of RFWD3 also exerted anti‐cancer efficacies in models of other cancers.^[^ ^18^, ^60^, ^61^ ^]^ Therefore, RFWD3 represents an attractive therapeutic target. Its inhibition—through small molecule inhibitors or proteolysis‐targeting chimeras (PROTACs)—may unleash STING‐IFN signaling within cancer cells. To validate this strategy, both preclinical and clinical studies are warranted, accompanied by vigilant monitoring for potential side effects, including hematological and gastrointestinal toxicities.

## 4 Experimental Section

### Patient Samples

The study was approved by the research ethics committee of the Cancer Hospital, Chinese Academy of Medical Sciences (NCC2020A190) and was conducted in accordance with the tenets of the Declaration of Helsinki. All samples were collected with informed consent. The diagnosis of NSCLC was confirmed by at least two pathologists. Formalin‐fixed, paraffin‐embedded tissue sections (5 µm) were obtained from the Department of Pathology of our hospital, and tissue microarrays were supplied by Xinchao Biotech (Shanghai). Details of patient demographics and clinical information are provided in Table 1.

### Chemicals and Reagents

The cigarette smoke extract (CSE) was prepared according to a modified Carp and Janoff protocol.^[^ ^62^ ^]^ In brief, non‐filtered cigarettes (2 units) were combusted using a smoke generation apparatus. The resulting smoke was passed through 50 mL of serum‐free culture medium (pH‐adjusted to 7.4) via bubbling. The medium was subsequently filtered through a 0.22 µm membrane to eliminate large particulate matter and bacterial contaminants. This stock solution was designated as 100% CSE and was appropriately diluted with fresh medium prior to experimental use.

PM_2.5_ was collected from four cities of China, including Beijing, Shanghai, Guangzhou, and Xi‐an on Teflon filters^[^ ^31^ ^]^ (diameter = 90 mm, Whatman, St. Louis, MO). The extraction process was performed following previously reported methods.^[^ ^63^, ^64^, ^65^ ^]^ The filter membrane samples containing PM_2.5_ were cut into 10 mm × 10 mm fragments and immersed in a 70% ethanol solution. Subsequently, the samples were sonicated in an ultrasonic homogenizer for 1 h to dissolve the PM_2.5_ particles. The resultant PM_2.5_ suspension was then freeze‐dried in a vacuum lyophilizer to obtain purified PM_2.5_ particles. Finally, the purified PM_2.5_ particles were dissolved in sterile PBS to prepare a stock solution with a concentration of 5 mg mL^−1^.

BaP (Cat: B1760), benzo(g,h,i)perylene (BzP, Cat: 380709), dibenzo(a,h)anthrancene (DbA, Cat: 48574), nicotine‐derived nitrosaminoketone (NNK, Cat: SY062201), and N′‐nitrosonornicotine (NNN, Cat: N075) were obtained from J&K Scientific (Beijing, China). Nicotine solution (LN3, 1 mg mL^−1^, Cat: N551), vinyl chloride solution (VC, 200 µg mL^−1^, Cat: 48625), and ethylene oxide (EO, 500 µg mL^−1^, Cat: CRM44609) were obtained from Sigma–Aldrich (St. Louis, MO, USA). Polystyrene (PS, diameter 0.6–1.0 µm, 1.06 g mL^−1^, Cat: L815938) was obtained from MACKLIN (Shanghai, China). BaP, BzP, NNK, NNN, and DbA were dissolved in dimethyl‐sulfoxide (DMSO, Cat: 472301, Sigma–Aldrich) to a stock concentration of 50 mM and stored at −20 °C. All compounds were diluted in culture medium to a final concentration of 10 µM and used to treat cells for 48 h.

### Cell Culture

Embryonic kidney HEK293T and lung adenocarcinoma cell lines A549, NCI‐H1975, large cell lung cancer line NCI‐H460, NCI‐H1299, lung squamous cell carcinoma cell lines NCI‐H520, and mouse‐derived cell line LLC were obtained from the American Tissue Culture Collection (ATCC, Manassas, VA, USA). Human bronchial epithelial cell line 16HBE was sourced from Clonetics (Walkersville, MD). BEAS‐2B bronchial epithelial cells were kindly provided by Professor Hongbin Ji at the Shanghai Institute for Biological Sciences, Chinese Academy of Sciences. The cells were cultured in Roswell Park Memorial Institute (RPMI) 1640 medium or Dulbecco Modified Eagle Medium (DMEM) supplemented with 10% fetal bovine serum (FBS, Gibco, Grand Island, NY, USA), 100 U mL^−1^ penicillin, and 100 µg mL^−1^ streptomycin in a humidified incubator at 37 °C with 5% CO2. Transient transfection of plasmids or siRNAs was conducted using the Lipofectamine 3000 kit (Invitrogen) according to the vendor's instructions.

### Plasmid Construction and Transfection

For constructing Flag‐, HA‐, and Myc‐tagged mammalian expression plasmids, cDNA of *RFWD3*, *TREX1*, or *TRIM24* was amplified using 2× Phanta Max Master Mix (Vazyme; Nanjing, Jiangsu, China) and inserted into pCDH‐CMV‐GFP, pcs2‐HA, or pcDNA3.1 vector. Transient transfection of plasmids or siRNAs was conducted using the Lipofectamine 3000 kit (Invitrogen; Frederick, MD, USA) according to the manufacturer's instructions. All primers, siRNAs, and shRNAs were synthesized by GenePharma (Shanghai, China) and listed in Table S2 (Supporting Information).

### Virus Packaging and Stable Cell Line Generation

Full‐length cDNA encoding mouse RFWD3 was amplified and inserted into a pCDH‐CMV‐GFP expression vector. Lentivirus particles were produced by co‐transfecting pCDH‐CMV‐RFWD3 into HEK293T cells with packaging plasmids pMD2.G and psPAX2. The indicated cells were infected with lentiviral particles expressing RFWD3 or an empty vector for 48 h, followed by subsequent experiments.

To generate knockdown cells, a pLKO.1‐puro based lentivirus was employed expressing specific short hairpin RNAs (shRNAs) against the target gene. H1299, A549, or LLC cells were infected with lentiviruses targeting RFWD3, STING, TREX1, or a negative control shRNA, and then selected with puromycin. Knockdown efficiency was determined using western blotting. The sequences for shRNAs were listed in Table S2 (Supporting Information).

### ELISA Assays

For determining IFNβ concentrations, culture supernatants were collected and measured using the human IFNβ ELISA Kit (Elabscience, Wuhan, Hubei, China). Quantification of IFNβ concentration was performed in triplicate according to the manufacturer's protocol, and the reading was obtained at 450 nm using a microplate reader and calculated using an IFNβ standard curve. The TRIM24‐binding capacity of TREX1 incubated with different concentrations of RFWD3. Plates coated with recombinant GST‐TRIM24 protein (0.5 µg mL^−1^) were incubated with recombinant His‐TREX1 protein (3 µg mL^−1^) and indicated different concentrations of RFWD3 protein. The binding between plate‐coated TREX1 and TRIM24 was measured by using HRP‐conjugated anti‐His tag antibody. The percentage of binding competition by RFWD3 was determined. Graphs shown are representative of at least two independent experiments.

### RNA Extraction and qPCR

Total RNA was extracted from cells using TRIzol reagent (Invitrogen), and cDNA was synthesized using the HiScript III First‐Strand cDNA Synthesis Kit (Vazyme).^[^ ^66^ ^]^ qRT‐PCR was performed using the SYBR Green Master Mix (Thermo Fisher, Basingstoke, UK). The 2‐ΔΔCt method was used to calculate relative levels of gene expression, and the relative mRNA level for each gene was normalized to the GAPDH. Data are shown as the relative abundance of mRNA compared to that in the control groups. All samples were assayed in triplicate.

### Western Blotting

For western blotting, cells were washed twice with cold PBS, and whole‐cell proteins were extracted in lysis buffer (40 mM Tris pH8.0, 150 mM NaCl, 1% NP40, 0.5% sodium deoxycholate, 0.1% SDS, and 2 mM sodium orthovanadate) containing a phosphatase inhibitor cocktail. Protein concentrations were normalized using a protein assay dye reagent. Samples were boiled at 95 °C for 15 min and subjected to SDS‐PAGE. After transfer, the membranes were blocked with 5% milk in TBST buffer (0.1% Tween 20) for 1 h. Primary antibodies (Table S3, Supporting Information) were diluted and incubated at 4 °C overnight. After washing three times with TBST buffer, goat anti‐rabbit or anti‐mouse secondary antibodies and HRP conjugates were incubated with the membranes for 1 h. After washing three times, membranes were covered with the Amersham ECL Select reagent and imaged using the ChemiDoc MP imaging system. Antibodies used in this study are listed in Table S3 (Supporting Information). The band intensities were quantified via densitometry analysis and normalized to Actin using ImageJ software.

### Co‐Immunoprecipitation and GST Pull‐Down

Cell lysates used for the IP assay were extracted in IP lysis buffer [40 mM Tris·Cl (pH 7.4), 137 mM NaCl, 1.5 mM MgCl_2_, 0.5% nonidet P‐40, 2 mM EDTA, and 1 mM PMSF, 1 mM proteinase inhibitor cocktail] at 4 °C for 30 min. Supernatants were clarified by centrifugation at a speed (12000 × g) for 15 min at 4 °C. The supernatants were incubated with the corresponding antibodies (2 µg) as indicated and 20 µL of Protein A/G PLUS‐Agarose (Santa Cruz Biotechnology, Santa Cruz, CA, USA) at 4 °C overnight. The beads were washed four times with IP lysis buffer. The beads were boiled in 2× sodium dodecyl sulfate (SDS) loading buffer for 15 min. The input lysates and immunoprecipitates were separated by SDS‐PAGE and western blotting.

Recombinant GST‐RFWD3 protein was synthesized by Wuhan Max Biotechnology Co., Ltd. His‐TREX1 is a commercial protein purchased from Cloud Clone. GST or GST‐RFWD3 proteins (15 µg) were incubated with Glutathione Sepharose 4B for 5 h at 4 °C, followed by incubation with His‐TREX1 at 4 °C overnight. The samples were then washed four times with lysis buffer. Finally, the agarose beads were boiled in SDS loading buffer for 15 min and analyzed using western blotting.

### Immunofluorescence Staining and Confocal Microscopy

The cells were seeded on cover slides, fixed with 4% formaldehyde for 30 min, and permeabilized with 0.5% Triton X‐100 at room temperature for 20 min. The cells were blocked with 5% bovine serum albumin (BSA) for 30 min. Then incubated with primary antibodies overnight at 4 °C, followed by incubation with secondary antibodies for 1–1.5 h at room temperature. After incubation with DAPI (Sigma, St. Louis, MO, USA) to stain the nuclei, the cells were visualized with a confocal laser scanning microscope N‐STORM Super‐Resolution (Nikon).

### Proximity Ligation Assay (PLA)

PLA was performed according to the Duolink in situ fluorescence protocol (Sigma–Aldrich). Briefly, cells were grown on glass coverslips, fixed with 4% paraformaldehyde for 30 min, and permeabilized with 0.5% Triton X‐100 for 20 min. The cells were blocked for 60 min at 37 °C with Duolink Blocking Solution, and the blocking solution was then replaced with the primary antibodies at 4 °C overnight. The subsequent step was incubating cells with the Duolink in situ PLA Probe Anti‐Rabbit PLUS and Anti‐Mouse MINUS PLA probes for 90 min at 37 °C. The cells were then incubated with ligation solution for 60 min at 37 °C and amplification solution for 100 min at 37 °C. Finally, the nuclei were stained with DAPI.

### Quantification of Cytosolic dsDNA

Approximately 1 × 10^7^ cells were lysed, and nuclear, cytosolic, and mitochondrial fractions were obtained using a mitochondrial isolation kit (Thermo Fisher). No protease inhibitors were used for subsequent DNA purification. The mitochondria were purified at 12000 × g to minimize contamination of the cytosolic fraction. DNA was subsequently isolated from the cytosolic fractions using the Qiagen DNeasy blood and tissue kit (Qiagen), and dsDNA was quantified using Qubit 2.0 (Invitrogen) and Qubit dsDNA HS Reagent.

### Flow Cytometry

To analyze tumor‐infiltrating immune cells, tumors were weighed, minced, and incubated with digestion buffer 0.5 mg mL^−1^ type IV collagenase (Thermo Fisher Scientific) for 45–60 min at 37 °C. Digestion was then terminated with medium, and the isolated tumor cells were passed through a 70 µm cell filter. After washing three times with PBS containing 2% inactivated FBS, the cell suspensions were blocked with an anti‐CD16/32 antibody (BioLegend, San Diego, CA; Table S3, Supporting Information). For immune cell activation and cytokine analysis, cells were resuspended in 1 mL of culture medium and stimulated with 2 µL of Cell Activation Cocktail (containing Brefeldin A to block cytokine secretion) for 4 h in a cell culture incubator, followed by two washes with PBS. Cell viability was assessed using Zombie dye (1:1000 dilution, 15–20 min incubation). Surface marker staining was performed using antibodies, including APC/Cy7‐anti‐mouse‐CD45 (pan‐leukocyte marker), FITC‐anti‐mouse‐CD3, PE/Cy7‐anti‐CD4, BV650‐anti‐mouse‐CD25, and Percp/Cy5.5‐anti‐mouse‐CD8 (T cell subsets), FITC‐anti‐mouse‐NK1.1 (NK cells), BV785‐anti‐mouse‐CD11c and BV421‐anti‐mouse‐MHC II (DC), as well as APC‐anti‐mouse‐CD11b and PE‐anti‐mouse‐GR1 (MDSC). After surface staining for 30 min on ice, cells were fixed and permeabilized by Fixation Buffer Set (BioLegend) for 1 h on ice and then intracellularly stained with antibodies for 30 min on ice, including: APC‐anti‐mouse‐IFN‐γ, PE‐anti‐mouse‐Granzyme B, and BV421‐anti‐mouse‐TNF‐α to evaluate cytokine production. Flow cytometry was performed using a flow cytometer and analyzed using FlowJo v10.8.1.

### 3′ to 5′ Exonuclease Activity Assay

The 3′ to 5′ exonuclease activity was measured using Exonuclease Activity Assay Kit (Abcam). Exonuclease activity was quantified in triplicate, according to the manufacturer's protocol. It provides a quick and easy method for monitoring 3′ exonuclease activities in various samples. In this assay, the exonuclease digests the provided DNA probe, producing a strong fluorescent signal (Ex/Em = 304/369 nm). The kit is simple, sensitive, high‐throughput, adaptable, and can detect exonuclease activity as low as 0.2 µU of exonuclease activity.

### Identification of TREX1‐Interacting Proteins by Mass Spectrometry

A549 cells were transfected with Flag‐tagged TREX1 or an empty vector for 48 h, cells were lysed in IP buffer (40 mM Tris‐HCl, pH 7.4, 150 mM NaCl, 1.5 mM MgCl_2_, 0.5% nonidet P‐40, 2 mM EDTA, and 1 mM PMSF, 1 mM proteinase inhibitor cocktail), followed by centrifugation at 12 000×g for 15 min at 4 °C. The supernatants were subjected to immunoprecipitation using anti‐Flag M2 magnetic beads (Sigma–Aldrich) for 10 h at 4 °C. The beads were washed five times with IP buffer. The eluted proteins were resolved by SDS‐PAGE, Coomassie brilliant blue stained, and the gel bands of proteins were excised for in‐gel digestion, and the proteins were identified using MS. Briefly, proteins were treated with 25 mM DTT and alkylated with 55 mM iodoacetamide to reduce disulfide. Sequencing grade modified trypsin in 50 mM ammonium bicarbonate was used for in‐gel digestion at 37 °C overnight. The peptides were extracted twice with 1% trifluoroacetic acid in a 50% acetonitrile aqueous solution for 30 min. To reduce the volume, the peptide extracts were centrifuged in a SpeedVac. For LC‐MS/MS analysis, peptides were separated by 60 min gradient elution at a flow rate of 0.3 µL min^−1^ with a Thermo‐Dionex Ultimate 3000 HPLC system, which was directly interfaced with a Thermo LTQ‐Orbitrap Velos Pro mass spectrometer.

### Multiplex Immunohistochemistry and Multispectral Imaging

mIHC assays were conducted to detect the cellular localization and expression levels of RFWD3, TREX1, dsDNA, CD8, and Granzyme B by using the PANO 4‐plex IHC kit (Panovue, Beijing, China) according to the manufacturer's recommendations. Specimens were incubated with anti‐RFWD3 (YT4064, Immunoway, San Jose, CA), anti‐TREX1 (185228, Abcam), anti‐dsDNA (270732, Abcam), anti‐CD8 (217344, Abcam), and anti‐Granzyme B (#46890, Cell Signaling Technology) antibodies, and TSA visualization and signal amplification were performed with Opal TSA Plus. The multiplex staining process ended with DAPI staining of cell nuclei, and the slides were covered by anti‐fluorescence‐quenching sealing agent and coverslips.

Slides were scanned and visualized by the PerkinElmer Mantra Quantitative Pathology Imaging System. The unmixing of multispectral images and the calculation of staining‐positive cell densities, which were expressed as the average number of positive cells per square millimeter, were performed by Perkin Elmer in Form Image Analysis software.

### Animal Models

Animal studies were approved and conducted in accordance with the Ethics Committee of Cancer Hospital, Chinese Academy of Medical Sciences (NCC2024A182). Six‐ to eight‐week‐old female C57BL/6J mice were purchased from Vital River Laboratory Animal Technology Co., Ltd., and maintained in a specific pathogen‐free environment for use in animal experiments. After 7 days of acclimatization, mice were randomly assigned into three groups (n = 5/group) and subcutaneously injected in the dorsal flanks with 1 × 10⁶ LLC cells transfected with shNC, sh*Rfwd3*‐#1, or sh*Rfwd3*‐#2. To investigate *RFWD3*’s effects on tumor growth, 2 × 10⁶ LLC cells expressing PCDH‐NC or PCDH‐*Rfwd3* were subcutaneously injected into C57BL/6J mice (n = 7/group). For the pulmonary metastasis model, 2 × 10^5^ LLC cells expressing shNC, sh*Rfwd3*‐#1, or sh*Rfwd3*‐#2 were intravenously injected via tail vein into C57BL/6J mice (n = 5/group).

To determine immune cell dependency in *RFWD3*‐mediated tumor suppression, immunodeficient NSG or *Rag1* ^−/−^ mice were randomly assigned into three groups (n = 5/group) and subcutaneously injected in the dorsal flanks with LLC cells transfected with shNC, sh*Rfwd3*‐#1, or sh*Rfwd3*‐#2. To assess CD8⁺ T cell dependency, sh*Rfwd3* LLC tumor‐bearing C57BL/6J mice (n = 6/group) received intraperitoneal (i.p.) injections of 100 µg anti‐mouse CD8α monoclonal antibody (BP0090; Bio X Cell) or IgG control every three days. For IFN signaling dependency, sh*Rfwd3* LLC tumor‐bearing C57BL/6J mice (n = 6/group) received (i.p.) injections of 100 µg anti‐mouse IFNAR1 monoclonal antibody or IgG control every three days. To evaluate synergy with PD‐L1 blockade, C57BL/6J mice bearing shNC and sh*Rfwd3* LLC tumors were randomized one week post‐inoculation into four groups: shNC + IgG, shNC + αPD‐L1, sh*Rfwd3* + IgG, and sh*Rfwd3* + αPD‐L1 (n = 6/group). Mice received (i.p.) injections of anti‐PD‐L1 monoclonal antibody or IgG control (100 µg/mouse). All procedures used randomized group assignment under specific pathogen‐free conditions. Tumor growth was monitored throughout using caliper measurements.

### Luciferase Assay

The promoter region of RFWD3, ‐2000 upstream of the initiating ATG codon, was amplified by PCR and cloned into the pGL3‐promoter plasmid. The cells were transfected with plasmids containing RFWD3 promoter‐driven luciferase. Luciferase activity was measured using the Dual luciferase reporter assay system (Promega, Madison, WI, USA).

### Statistical Analysis

All experiments were performed at least three times, and the results are presented as the mean ± standard deviation (SD). Differences between groups were evaluated for significance using a two‐tailed Student's *t*‐test or a Two‐way ANOVA test. The log‐rank (Kaplan–Meier) test was used for survival rate analysis. In all figures, ^*^, *p*< 0.05; ^**^, *p*< 0.01; ^***^, *p*< 0.001; ^****^, *p*< 0.0001; ns, not significant.

## Conflict of Interest

The authors declare no competing interests.

## Author Contributions

X.‐Y. S. and Y.‐K.S. contributed equally to this work. G.‐B.Z. conceived the project. X.‐Y.S. designed and performed the study. Y.‐K.S., Y.S., M.‐Y.L., Y.‐F.L., Z.W., Z.L., X.‐L.J., and G.‐Z.W. performed the study. X.‐Y. S., Z.R., and G.‐B.Z. analyzed the results and wrote the manuscript. Y.‐X.F. provided RAW cells and *Rag1* KO mice. All authors discussed the results and reviewed the manuscript.

## Supporting information



Supporting Information

Supporting Information

## Data Availability

The data that support the findings of this study are openly available in TCGA at www.cghub.ucsc.edu/, reference number ^[^ ^29^ ^]^
