# Supplementary material for: Smohaze‐Upregulated RFWD3 Competes with TRIM24 to Stabilize TREX1 and Reduce Cytosolic dsDNA in Non‐Small Cell Lung Cancer
Source: Adv Sci (Weinh). 2025 Oct 21;12(48):e08481. doi: 10.1002/advs.202508481 (PMC12752551; doi:10.1002/advs.202508481)
Supplement: Supplementary file 1 — Supporting Information [file ADVS-12-e08481-s001.docx]

**Supplementary information for**

**Smohaze-Upregulated RFWD3 Competes with TRIM24 to Stabilize TREX1 and Reduce Cytosolic dsDNA in Non-small Cell Lung Cancer**

Xue-Yan Shi^1,3*^, Yu-Ke Shen^1,2*^, Meng-Yao Lv^1^, Yu Sun^4^, Yong-Fang Lin^1^, Zheng Wang^1^, Xiao-Liang Jie^1^, Zheng Liu^1^, Yang-Tong Liu^1^, Yang-Xin Fu^4,5^, Zhenhua Ren (✉)^4,6^, Gui-Zhen Wang (✉)^1^, Guang-Biao Zhou (✉)^1, 2^

*These authors equally contributed to this work.

Correspondence to: Guang-Biao Zhou ([gbzhou@cicams.ac.cn](mailto:gbzhou@cicams.ac.cn)) or Gui-Zhen Wang ([gzwang@cicams.ac.cn](mailto:gzwang@cicams.ac.cn)) or Zhenhua Ren ([renzhenhua@cpl.ac.cn](mailto:renzhenhua@cpl.ac.cn)).

**This file contains eight supplementary figures and two supplementary tables.**

**Table S2. Sequences of siRNA, shRNA, and primers used in this study.**

| Target | Sequence (5′-3′) |
| --- | --- |
| **siRNAs** |  |
| si*RFWD3*-1 | GGACCUACUUGCAAACUAU |
| si*RFWD3*-2 | AACUCCUGCACAUGACUGC |
| si*TREX1* | GCUAUAGCCUAGGCAGCAUTT |
| si*HSP90AB1*-1 | CAGUGGUAAAGAGCUGAAATT |
| si*HSP90AB1*-2 | GAAGCAUUCUCAGUUCAUATT |
| si*DHX15*-1 | GGAGCGUUAUGGUGUAAUATT |
| si*DHX15*-2 | GCAGCAAUUCGAACAGUUATT |
| si*RBM10*-1 | CCAACUGAACCGCGGCUUUTT |
| si*RBM10*-2 | GGACAUGGCCUCCAAUGAATT |
| si*IPO7*-1 | CGCCAUUGUAUUCGAGAAATT |
| si*IPO7*-2 | GUCCAUUGGUAGCAGCAAUTT |
| si*MYH9*-1 | CCGAUAAGUAUCUCUAUGUTT |
| si*MYH9*-2 | GAUCAAUCCAUCUUGUGCATT |
| si*SF3B1*-1 | CCUCGAUUCUACAGGUUAUTT |
| si*SF3B1*-2 | GCACAGACCUCCAAAGAUUTT |
| si*KRT1*-1 | CUCCACUAGAACCCAUAAUTT |
| si*KRT1*-2 | GAGUCUUGUUAACCUUGGUTT |
| si*KRT9*-1 | CUCUUCUAGUGGCUAUGGUTT |
| si*KRT9*-2 | GACAAUCUGACCAUGGAGATT |
| siALB-1 | GGUAACCUUUAUUUCCCUUTT |
| siALB-2 | GCUCAUCGGUUUAAAGAUUTT |
| **shRNAs** |  |
| Human sh*RFWD3*-1 | GGACCUACUUGCAAACUAU |
| Human sh*RFWD3*-2 | GUUAAGAUGUUGAGUACUG |
| Mouse sh*RFWD3*-1 | GCAACAGACCAAGAAGCTACA |
| Mouse sh*RFWD3*-2 | GGTGAAACTTGCACAATATGT |
| Mouse sh*STING* | GCCATACTCCAACCTGCATCC |
| Mouse sh*TREX1* | GCTACCACTGGAACAACCAAC |
| **Primers** |  |
| *HSP90AB1* | F:AAGAGAGGCGGGTCAAAGAA  R:CGCTGTCATCCTCCTCATCT |
| *DHX15* | F:TGGTTCAGTTGCATCCCTCT  R:CGGTCCAACTGTCTCTTTGC |
| *RBM10* | F:AAGTGCTTCAAATGTGGCGT  *R:AGGTTGCGCAAAATGATGGT* |
| *IPO7* | F:AAGTGCTTCAAATGTGGCGT R:AGGTTGCGCAAAATGATGGT |
| *MYH9* | F:AAGTGCTTCAAATGTGGCGT R:AGGTTGCGCAAAATGATGGT |
| *SF3B1* | F:ACGTGACATCAATTGCTGCA R:TCTTCCCGGTCTGCAATCTT |
| *RFWD3* | F:AGGTTGAGAGCACCATTGGA R:ATTCCTCTGCCTGGTCAACA |
| *TREX1* | F:ACCATCTGCTGTCACAACCACTG R:ATAGGGCTCCAGGGTCCTTCAC |
| *KRT 1* | F:AGAGTGGACCAACTGAAGAGT R:ATTCTCTGCATTTGTCCGCTT |
| *KRT9* | F:GGGGCCGATTCAGCTCTTC  R:CTACTGGCACTAAAACCACCC |
| *ALB* | F:GAGACCAGAGGTTGATGTGATG R:AGTTCCGGGGCATAAAAGTAAG |
| *IRF3* | F:AGAGGCTCGTGATGGTCAAG R:AGGTCCACAGTATTCTCCAGG |
| *STAT1* | F:CGGCTGAATTTCGGCACCT R:CAGTAACGATGAGAGGACCCT |
| *ISG15* | F:CGCAGATCACCCAGAAGATCG R:TTCGTCGCATTTGTCCACCA |
| *CCL5* | F:CCAGCAGTCGTCTTTGTCAC  R:CTCTGGGTTGGCACACACTT |
| *CXCL10* | F:GTGGCATTCAAGGAGTACCTC  R:TGATGGCCTTCGATTCTGGATT |
| *RFWD3 P1* | F:TGTTTGAAGCAATTATAG  R:CTGAATGGACAAGTGGAT |
| *RFWD3 P2* | F:TCAGTAGCTCAGATCCCT  R:AGAGGCGTAGGACAGTCC |
| *GAPDH* | F:CCAGGTGGTCTCCTCTGA  R:GCTGTAGCCAAATCGTTGT |

**Table S3. Antibodies used in this study.**

| **Antibody** | **Vendors** | **Catalog number** |
| --- | --- | --- |
| **Western blotting** |  |  |
| cGAS | Cell Signaling Technology | 83623 |
| p-STAT1 | Cell Signaling Technology | 7649 |
| STAT1 | Cell Signaling Technology | 9172 |
| p-TBK1 | Cell Signaling Technology | 5483 |
| TBK1 | Cell Signaling Technology | 3504 |
| p-IRF3 | Cell Signaling Technology | 37829 |
| IRF3 | Cell Signaling Technology | 4302 |
| p-STING | Cell Signaling Technology | 50907 |
| STING | Cell Signaling Technology | 13647 |
| RFWD3 | ELK | ES6711 |
| TREX1 | Abcam | 185228 |
| TRIM24 | Abcam | 70560 |
| AHR | Abcam | 314060 |
| β-actin | Proteintech | 66009-1-Ig |
| IgG | Proteintech | SA00001-4 |
| HA | BioLegend | 901501 |
| Flag | Sigma | f1804 |
| Ubiquitin | Cell Signaling Technology | 20326 |
| **Immunoprecipitation** |  |  |
| RFWD3 | Abcam | 99306 |
| TREX1 | Abcam | 238338 |
| HA | BioLegend | 901501 |
| Flag | Sigma | f1804 |
| IgG | Cell Signaling Technology | 93702 |
| **Multiplex immunohistochemistry** | | |
| RFWD3 | Immunoway | YT4064 |
| TREX1 | Abcam | 185228 |
| dsDNA | Abcam | 270732 |
| Granzyme B | Cell Signaling Technology | 46890 |
| **Immunofluorescence** |  |  |
| dsDNA | NOVUS | 07302 |
| RFWD3 | Immunoway | YT4064 |
| TREX1 | Abcam | 185228 |
| **Flow cytometry**  **Antibodies and fluorophore-conjugated** | | |
| Anti-mouse CD45, APC/Cy7 | BioLegend | 147707 |
| Anti-mouse CD3, FITC | BioLegend | 100203 |
| Anti-mouse CD25, BV650 | BioLegend | 102037 |
| Anti-mouse CD4, PE/Cy7 | BioLegend | 100421 |
| Anti-mouse CD8, PerCp/Cy5.5 | BioLegend | 155013 |
| Anti-mouse NK1.1, FITC | BioLegend | 156507 |
| Anti-mouse-CD11c, BV785 | BioLegend | 117335 |
| Anti-mouse MHC II, BV421 | BioLegend | 107631 |
| Anti-mouse CD11b, APC | BioLegend | 101211 |
| Anti-mouse GR1, PE | BioLegend | 108407 |
| Anti-mouse IFN-γ, APC | BioLegend | 505809 |
| Anti-mouse Granzyme B, PE | BioLegend | 372207 |
| Anti-mouse TNF-α, BV421 | BioLegend | 506327 |

**
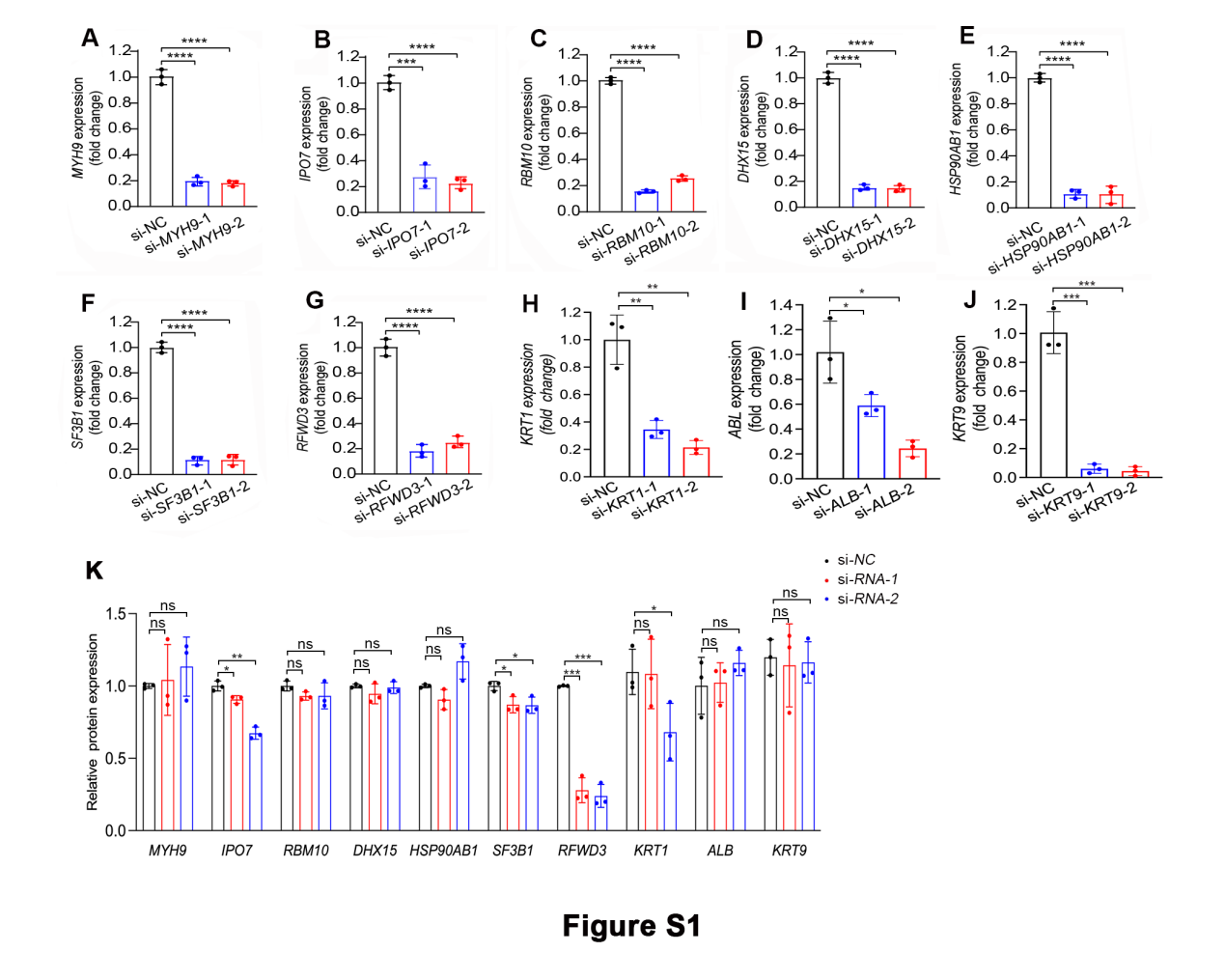
**

**Fig. S1. Transfection efficacy and TREX1 expression levels in cells transfected with siRNAs against indicated genes.**

(A-J) The expression levels of *MYH9*, *IPO7*, *RBM10*, *DHX15*, *HSP90AB1*, *SF3B1,* *RFWD3, KRT1, ALB* and *KRT9* in A549 cells were determined by qPCR.

(K) A549 cells were transfected with different siRNAs and subjected to western blotting (Fig. 1B), followed by quantitative densitometry analyses of protein bands. Error bars, SD. *P*-values were calculated using a two-tailed Student's t-test. *, *P* < 0.05; **, *P* < 0.01; ***, *P* < 0.001; ****, *P* < 0.0001; ns, not significant.

**
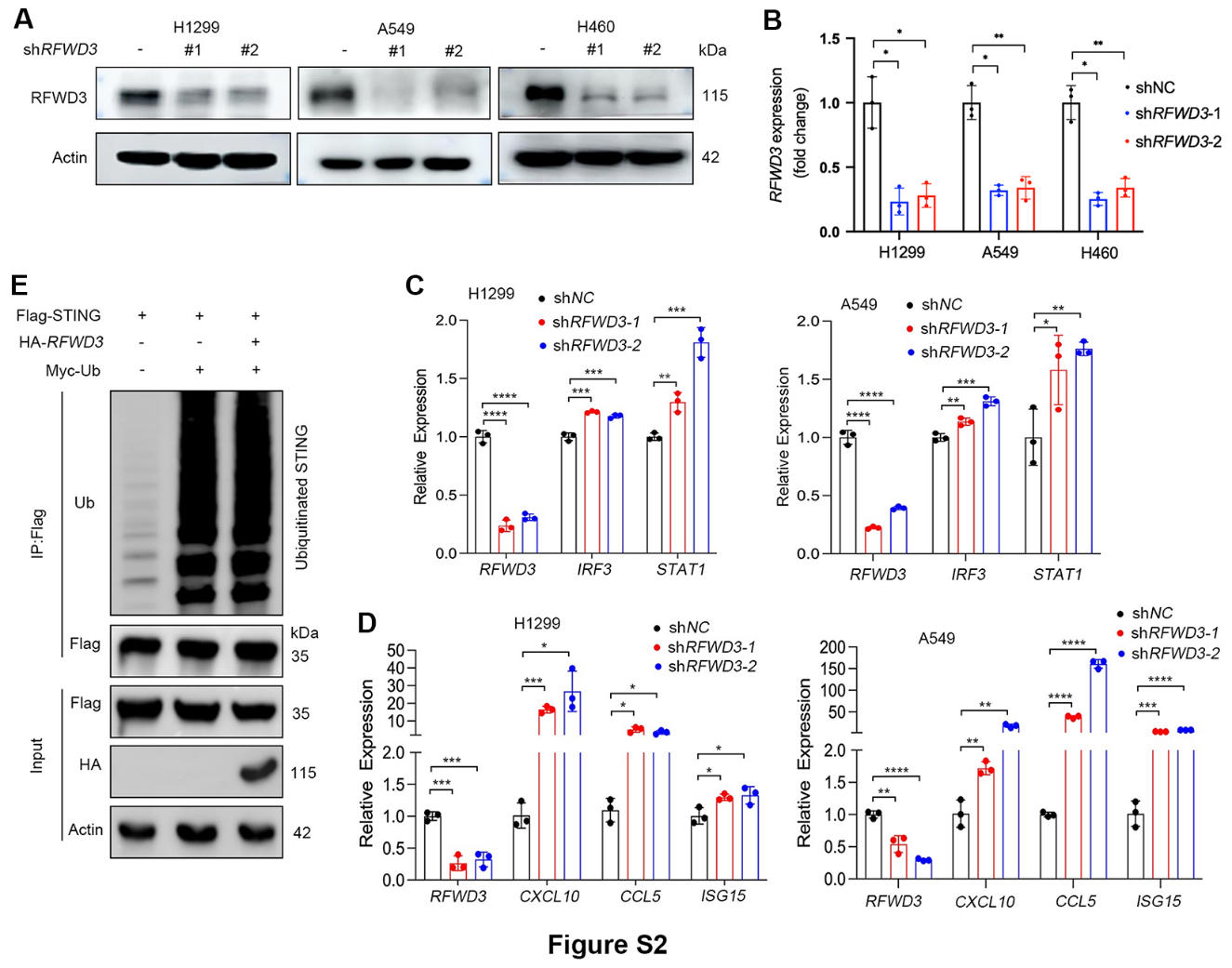
**

**Fig. S2. The impact of RFWD3 on STING signaling pathway activity and associated biological functions**

(A, B) Western blotting (A) and qPCR (B) verification of knockdown efficiency of both sh*RFWD3*-#1 and -#2 in H1299, A549, and H460 cells.

(C) H1299 and A549 cells were transfected with two different shRNAs against *RFWD3*, and the mRNA expression levels of *RFWD3*, *IRF3*, and *STAT1* were quantitatively detected by qPCR.

(D) H1299 and A549 cells were transfected with two different shRNAs against *RFWD3*, and the mRNA expression levels of *RFWD3*, *CXCL10*, *CCL5*, and *ISG15* were detected by qPCR.

(E) HEK293T cells were co-transfected with Flag-*STING*, HA-*RFWD3* and Myc-Ub for 48 h. Co-IP and immunoblotting were performed using cell lysates and indicated antibodies.

Error bars, SD. *P* Values were calculated using a two-tailed Student's t-test. *, *P* < 0.05; **, *P* < 0.01; ***, *P* < 0.001; ****, *P* < 0.0001.

**
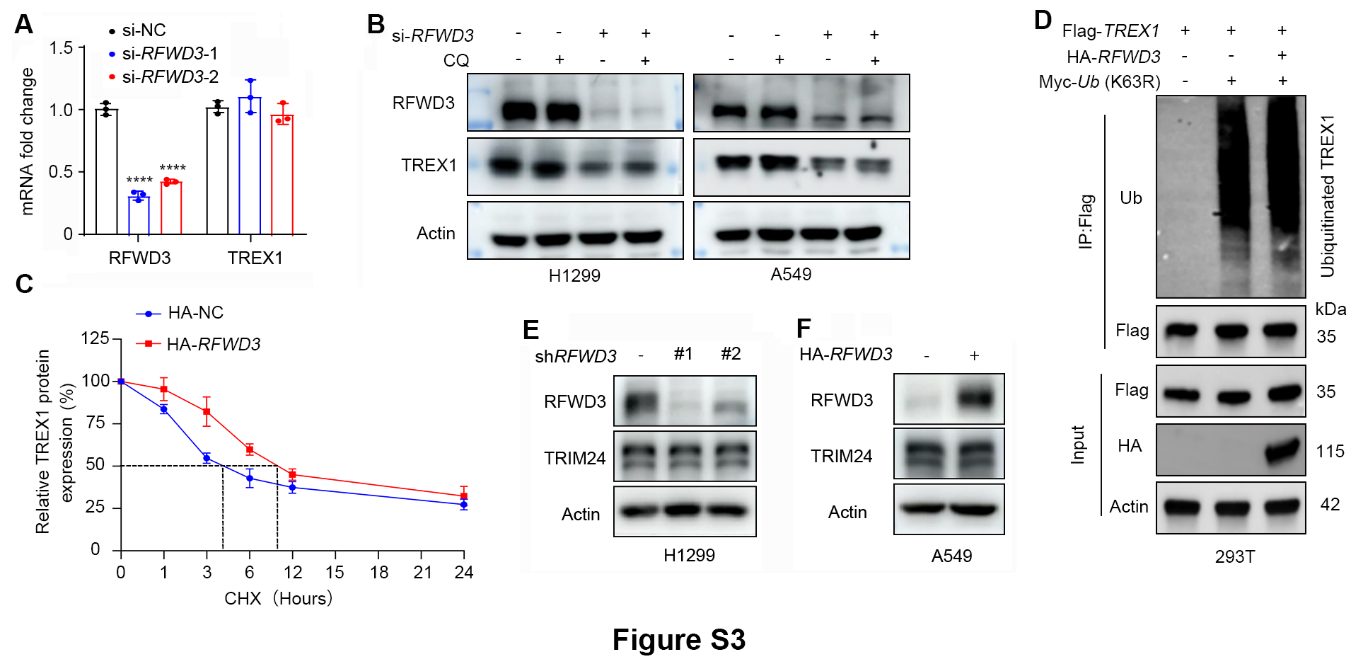
**

**Fig. S3. The impact of targeting RFWD3 on TREX1 and TIRIM24.**

1. A549 cells were transfected with si*RFWD3*, and subjected to qPCR.

(B) H1299 and A549 cells were transfected with si*RFWD3* for 48 h, treated with Chloroquine (CQ) for 24h, lysed, and subjected to western blotting using indicated antibodies.

(C) A549 cells were transfected with HA-*RFWD3* and treated with CHX for different durations. The protein detection results from western blotting were subjected to densitometric scanning for quantitative analysis.

(D) HEK293T cells were co-transfected with Flag-*TREX1*, HA-*RFWD3* and Myc-*K63R* for 48 h. Co-IP and immunoblotting were performed using the indicated antibodies and cell lysates.

(E) H1299 cells were transfected with two different sh*RFWD3* for 48 h, and subjected to western blotting.

(F) A549 cells were transfected with HA-*RFWD3* for 48 h, and subjected to western blotting.

Error bars, SD, *P* Values were calculated using a two-tailed Student's t-test. ****, *P* < 0.0001.


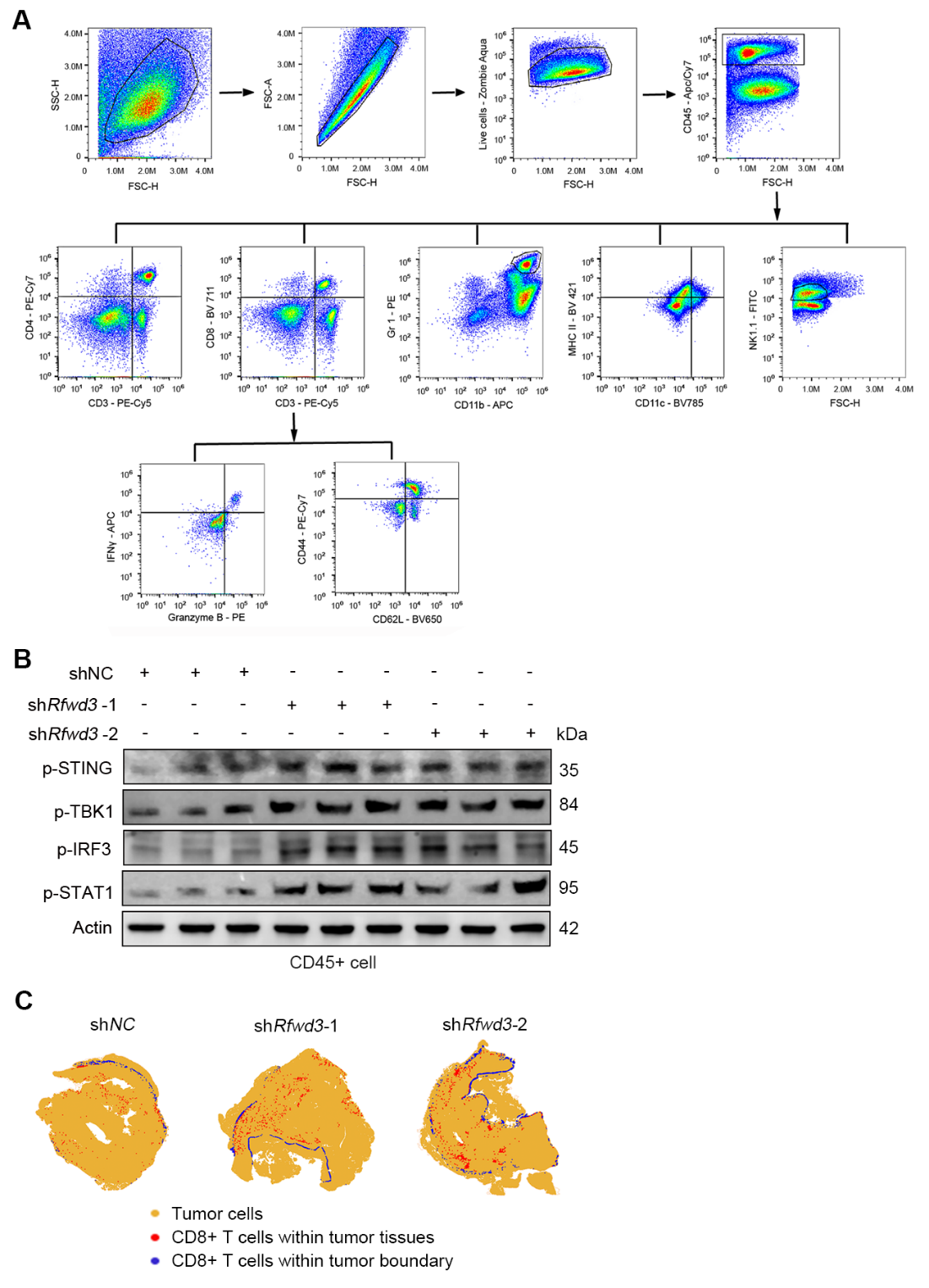


**Fig. S4. Effects of RFWD3 on tumor immune microenvironment.**

1. Representative gating strategy for determination of different immune cell populations.
2. LLC-NC and LLC-sh*Rfwd3* cells were subcutaneously transplanted into C57 mice, the tumors were resected 17 days later, CD45^+^ cells were sorted from the tumor tissue with flow cytometric cell sorting, and lysed for western blotting using indicated antibodies.
3. Spatial distribution of CD8^+^ T cells within subcutaneous tumors of mice inoculated with LLC cells expressing control or *Rfwd3*, as assessed by multiplex immunofluorescence analysis and HALO software.

**
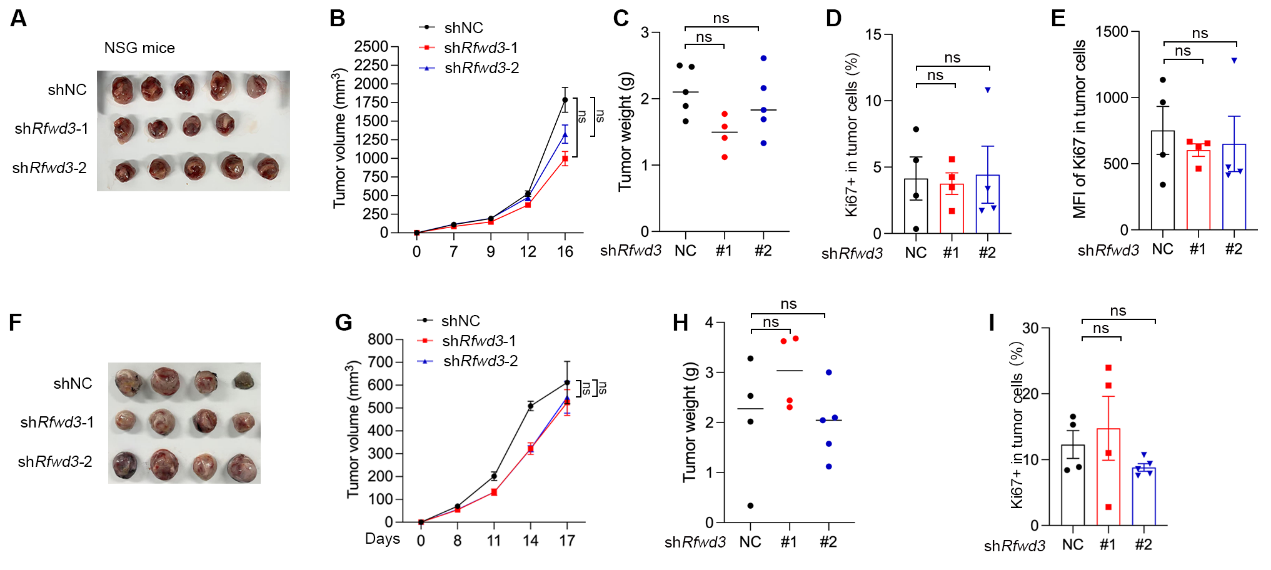
**

**Fig. S5. Effects of RFWD3 on tumor growth in immunodeficiency mice.**

(A) Tumors in NCG mice subcutaneously inoculated with LLC cells stably expressing control or sh*Rfwd3*.

(B, C) Tumor volume at indicated time points (B) and tumor weight at day 16 (C).

(D) The percentages of Ki67^+^ cancer cells in tumor tissues from NCG mice on 16 days after inoculation with LLC cells expressing control or *Rfwd3* shRNA, detected by flow cytometry.

(E) The mean fluorescence intensity (MFI) of Ki67 on cancer cells collected from tumor tissues of NCG mice.

(F) Tumor images in *Rag1* KO mice subcutaneously inoculated with LLC cells stably expressing control or sh*Rfwd3*.

(G, H) Tumor volume at indicated time points (G) and tumor weight at day 17 (H).

(I) The percentages of Ki67^+^ cancer cells in tumor tissues from *Rag1* KO mice on 16 days after inoculation with LLC cells expressing control or *Rfwd3* shRNA, detected by flow cytometry.

Error bars, SD. *P*-values were calculated using a two-tailed Student's t-test (C, D, E, H, I) or a Two-way ANOVA test (B, G). ns, not significant.

**
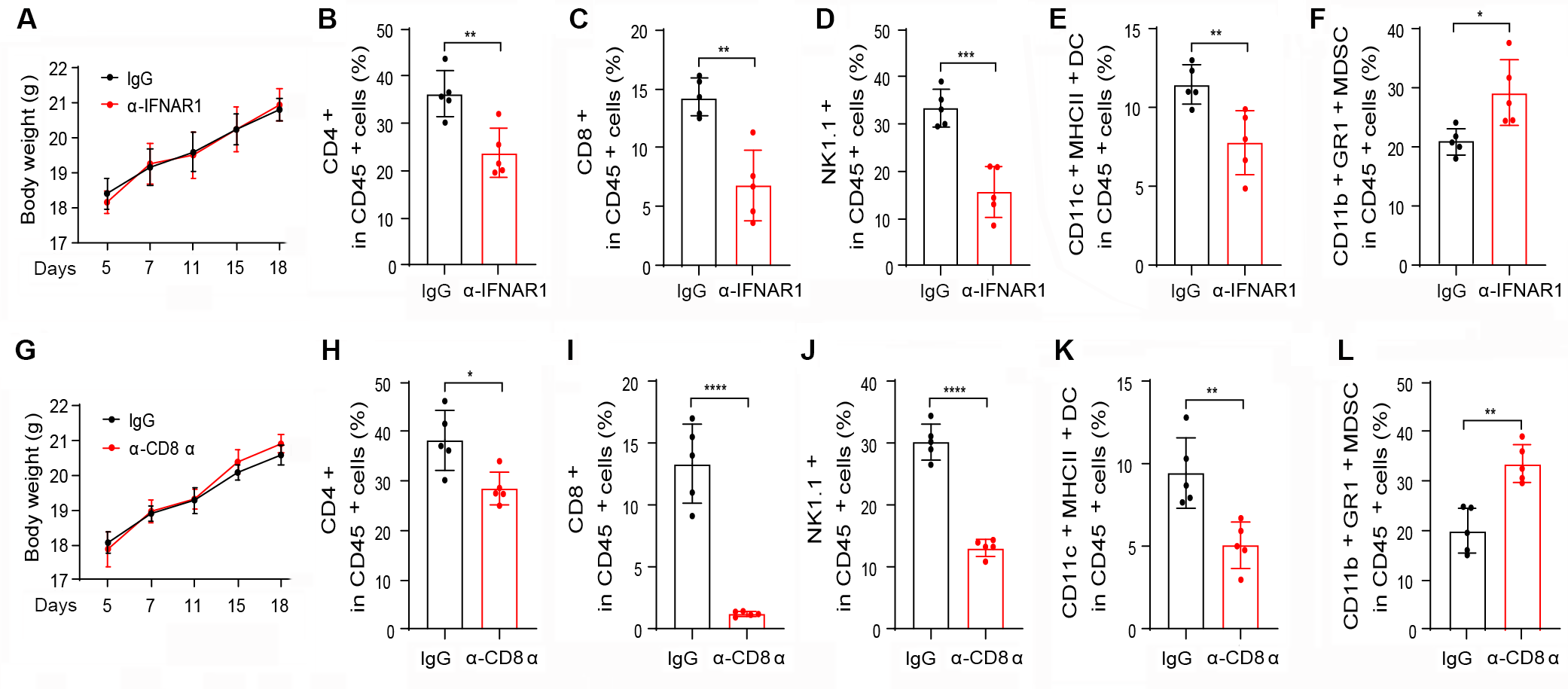
**

**Fig. S6. Host IFNAR1 pathway is essential for RFWD3-induced tumor progression.**

(A) Body weight of mice at indicated time points after the initiation of treatment with anti-IFNAR antibody and/or IgG antibody.

(B-F) Flow cytometry for the percentages of indicated types of tumor-inﬁltrating immune cells in tumor tissues taken from C57BL/6 mice 18 days after subcutaneous inoculation with LLC cells and treatment as described in (A).

(G) Body weight of mice at indicated time points after the beginning of treatment with anti-CD8α antibody and/or IgG antibody.

(H-L) Flow cytometry for the percentages of indicated types of tumor-inﬁltrating immune cells in the tumor tissues taken from C57BL/6 mice 18 days after subcutaneous inoculation with LLC cells and treatment as described in (G).

Error bars, SD. *P* Values were calculated using a two-tailed Student's t-test. *, *P* < 0.05; **, *P* < 0.01; ***, *P* < 0.001; ****, *P* < 0.0001.

**
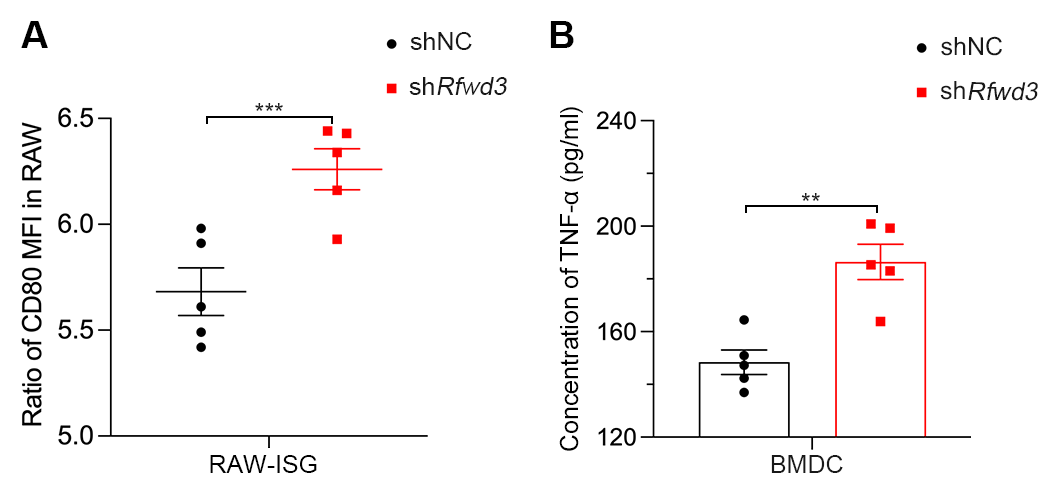
Fig. S7. Targeting RFWD3 activates tumor-associated immune cells.**

(A) The ratio of mean fluorescence intensity (MFI) of CD80 expression on RAW-ISG cells two days after RAW-ISG and LLC cells coculture.

(B) Concentration of TNF-α in the supernatant was measured by cytometric bead array two days after BMDC-OTI-LLC cells coculture.

Error bars, SD. *P* Values were calculated using a Two-way ANOVA test. **, *P* < 0.01; ***, *P* < 0.001.


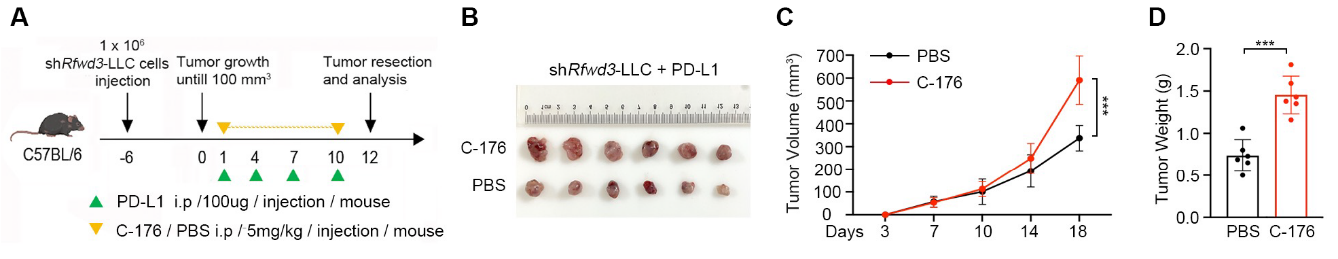


**Fig. S8. The synergistic effect between RFWD3 inhibition and PD-L1 blockade depends on STING.**

(A) Schematic of the experimental workﬂow. C57BL/6 mice subcutaneously inoculated with LLC cells stably expressing *Rfwd3* shRNA were injected with anti-PD-L1 antibody and C-176 or PBS as indicated.

(B) Images of resected tumors.

(C-D) Tumor volume at indicated time points (C) and tumor weight at day 18 (D).

Error bars, SD. *P* Values were calculated using a Two-way ANOVA (C) or a two-tailed Student's t-test (D). ***, *P* < 0.001.
